# Synergistic interactions in designed bacterial consortia: Dual effects on control rusty root rot and growth promotion in ginseng

**DOI:** 10.1016/j.synbio.2026.04.003

**Published:** 2026-06-03

**Authors:** Meng Tian, Yu Wang, Keying Sun, Lingxin Kong, Xiaolin Chen, Kaixin Zhang, Lina Yang, Baohui Lu, Jie Gao, Wanpeng Hou, Yongzhe An, Yun Jiang, Changqing Chen

**Affiliations:** a College of Plant Protection, Jilin Agricultural University, Changchun, 130118, China; b College of Life Science, Jilin Agricultural University, Changchun, 130118, China; c Jilin Provincial Ginseng Industry Development Service Center (Jilin Provincial Chinese Medicine Development Center), Changchun, 130033, China; d Jilin Shenwang Plant Protection Co., Ltd., Fusong, 134500, China

**Keywords:** Ginseng rusty root rot, Synthetic microbial community, Synergy mechanism, Biological control, Growth promotion

## Abstract

Ginseng rusty root rot, caused by *Ilyonectria robusta*, is the most severe soil-borne disease affecting ginseng cultivation. A synthetic microbial community (SynCom) was developed for the first time using antagonistic and growth-promoting endophytic and rhizosphere bacterial strains to control ginseng rusty root rot, and its synergistic mechanisms had been clarified. The 89 out of 98 biocontrol bacterial groups exhibited synergistic inhibitory effects against *I. robusta*, while 40 consortia showed both growth-promoting and synergistic effects. Among these, the 24 consortia inhibited mycelial growth of *I. robusta* by more than 80%, and seven consortia suppressed spore growth by over 60%. The inhibition rates were increased by 19%–131% and 5%−137%, respectively, compared with single strain treatment. The biocontrol consortia achieved superior control efficacy against ginseng rusty root rot in the field, which six consortia exhibited control efficacy exceeding 50%, with the consortium CL95 showing the highest efficacy at 66.67% and significantly promoted ginseng growth. The content of *I. robusta* in ginseng roots decreased by 46.97% following CL95 treatment compared to the single-strain NJ13. Mechanistic studies indicated that the colonization efficiency of the consortia in ginseng tissues and rhizosphere soil was significantly higher than that of single strains within 63 days. The colonization of CL95 in ginseng leaves, stems, epidermis, root core, lateral root, fibrous root and soil was 33%–215% higher than that of single strain NJ13, respectively. In addition, compared with NJ13, the four soil enzyme activities were increased by 100%–150%, and the expression levels of five defense enzyme genes in three different growth periods of ginseng were increased by 75%–167%, among which CL95 had the most obvious effect. It was also found that biocontrol consortia significantly increased the diversity of microbial communities and the abundance of beneficial fungi and bacteria in ginseng rhizosphere soil, while significantly reducing the abundance of pathogenic fungi. Correlation analysis showed that the consortia were significantly positively correlated with root weight and soil enzyme activity, promoting ginseng growth. This study lays a theoretical foundation for overcoming the limitations of single-strain biocontrol agents and highlight the potential for industrial application of biocontrol consortia in sustainable agriculture.

## Graphical abstract

**Figure:**
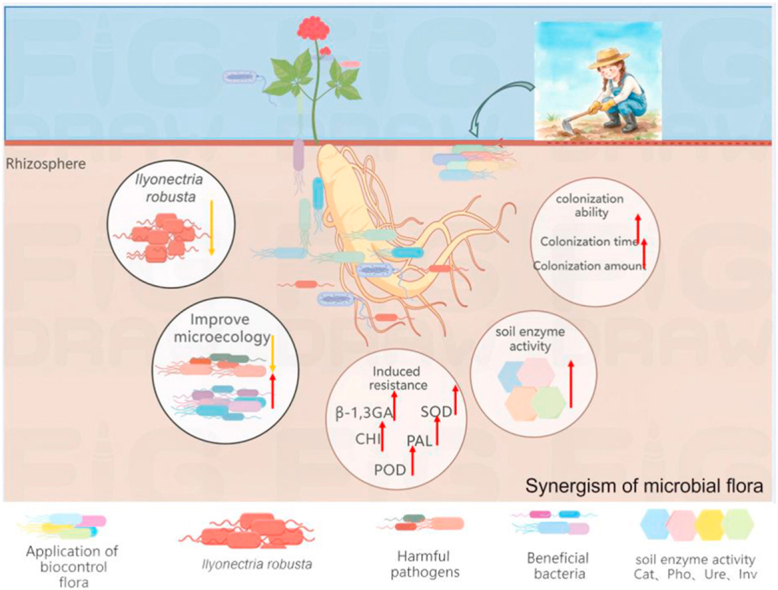

## 1 Introduction

While single-strain biocontrol agents (BCAs) have shown potential in suppressing plant pathogens, their practical application faces significant challenges. Monocultures often exhibit inconsistent field efficacy due to environmental sensitivity, limited colonization capacity, and narrow-spectrum activity against complex soil-borne pathogens [1]. For instance, in ginseng cultivation, single-strain BCAs struggle to combat *Ilyonectria destructans*, the causal agent of rusty root rot, owing to the pathogen's latent infection strategy and the perennial growth cycle of ginseng [2]. Furthermore, reliance on singular modes of control, such as antibiotic production or nutrient competition, increases the risk of pathogen resistance and fails to address multifactorial disease dynamics [3,4]. These limitations underscore the need for more effective and adaptable biocontrol strategies.

Synthetic microbial community (SynCom) offer a paradigm shift by harnessing synergistic interactions among diverse microorganisms to enhance biocontrol efficacy and functional redundancy. For example, combining *Pseudomonas fluorescens* T5 with non-antagonistic bacteria induced antifungal activity against *Rhizoctonia solani*, was significantly better than individual strains [5]. Similarly, SynCom have successfully suppressed soil-borne diseases like pea sclerotinia (*Sclerotinia sclerotiorum*) and tomato fusarium wilt (*Fusarium oxysporum*) by integrating complementary mechanisms: antibiosis, induced systemic resistance (ISR), and niche competition [5,6]. These consortia not only amplify antimicrobial metabolite production but also improve root colonization and resilience to environmental fluctuations, demonstrating superior adaptability compared to single-strain biocontrol agents (BCAs) [3,7]. Such successes highlight the potential of SynCom to address the complexity of plant-pathogen interactions. The microbial community induces plant ethylene reactions to control certain stages of lateral root development [8]. The combined use of strains can provide protection to help determine the characteristics associated with the function of microbiota in other biological systems [9]. The SynCom proliferates in the rhizosphere and reduces soil-borne diseases through the synergy of microorganisms, highlighting the potential of synergy between microorganisms in enhancing plant health [10]. The rhizosphere microbiome of resistant varieties enriched different and specific bacterial groups that were significantly associated with disease inhibition [11]. The compound population of *R. solanacearum* with allele markers was constructed artificially, and the ecological model was used to explore the population dynamics of *R. solanacearum* [12].

Despite growing interest in biocontrol for ginseng, progress remains hindered by the plant unique challenges including long cultivation cycles, soil degradation from monocropping, and the absence of registered microbial formulations targeting rusty root rot [2]. Existing studies on ginseng BCAs predominantly focus on single strain isolates with moderate efficacy, lacking the research and application of SynCom [1]. Future research should prioritize consortium design principles, strain compatibility, field validation and mechanism clarity. By bridging microecological theory and agricultural practice, SynCom could pave a novel way for sustainable ginseng cultivation, reducing reliance on chemicals and mitigating yield losses caused by rusty root rot.

The objective of this study was to artificially construct and screen synthetic microbial consortia using biocontrol endophytic and rhizobacterial strains isolated from ginseng, aiming to obtain microbial communities with enhanced control efficacy against ginseng rusty root rot and growth-promoting properties. Through comprehensive investigations of antimicrobial activity, plant growth promotion, colonization characteristics, induced systemic resistance, and soil microecological regulation, it elucidated the synergistic mechanisms of the microbial consortia. The findings are expected to establish a theoretical foundation for developing and applying high-efficiency biocontrol microbial communities.

## 2 Materials and methods

### 2.1 Strains and culture

The biocontrol bacterial strains NT35, NJ13, FG14, JA26, JA38, TU26, TJ23-2, JJ5-2, JI39-2, and JI6 were provided by the Green Prevention and Control Laboratory of Ginseng Disease in Jilin Agricultural University. Plant pathogenic fungal *Ilyonectria robusta* CBLJ-3 causing ginseng rusty root rot was cultured on potato dextrose agar (PDA) medium at 25 °C for 10 days, while biocontrol bacteria were cultured on Luria-Bertani (LB) agar medium at 28 °C for 2 days. The culture media of fermentation broth of the strains were shown in Table S1.

### 2.2 Strain compatibility assessment and consortium construction

Strain compatibility was evaluated using the agar block method. The measurement of two strains was summarized as an example. The fermentation broths of one bacterial strain was mixed with LB agar medium to create bacterial plates and another bacterial strain was inoculated as 8 mm agar plugs at the center of each bacterial plate. Plates were incubated at 28 °C for 2–3 days, and the presence of inhibition zone around the central colony was recorded to assess antagonistic interactions.

For consortium preparation, individual biocontrol strains were firstly cultured on LB solid medium at 28 °C for 24 h. Single colonies were transferred to LB liquid broth and incubated at 28 °C with shaking (180 rpm) to prepare seed cultures liquid, and then were inoculated into fermentation broth. Bacterial suspensions were standardized to an optical density (OD_600_) of 2.0 using sterile water. Subsequently, a combinatorial design was employed to mix standardized single-strain suspensions at a 1:1 ratio, resulting in a total of 98 distinct biocontrol consortium formulations (Table S3).

### 2.3 Determination of antagonistic and growth-promoting properties

#### 2.3.1 Antagonistic activity

The plate confrontation method was employed to evaluate antagonistic effects. Briefly, *I. robusta* CBLJ-3 was inoculated at the center of PDA plates. Subsequently, a 50 μL of single-strain or consortium fermentation broths were spot-inoculated 27 mm radially from the pathogen colony, with single culture of *I. robusta* CBLJ-3 as a control. Plates were incubated at 25 °C for 10 days, and pathogen inhibition was quantified by measuring mycelial diameter using the cross-measurement technique once control plates were fully colonized. Three biological replicates were performed each treatment.

#### 2.3.2 In vitro evaluation of biocontrol strains/consortia against *I. robusta* in ginseng roots

Healthy 3-year-old ginseng roots (asymptomatic, uniform in size) are washed clean with tap water. The root samples were immersed in 70% ethanol (EtOH) to describe the surface disinfection of sodium hypochlorite for 5 min, rinsed with 2% sodium hypochlorite (NaClO) for 3 min, and finally rinsed with sterile distilled water. Each rinsing step is performed 3 times disinfection method described by Liu et al. [13]. Three 3-mm-deep wounds (2 mm diameter) were created at equidistant sites on each ginseng root using a sterile pipette tip. At each wound site, 10 μL of biocontrol strain/consortium fermentation broth (1 × 10^8^ CFU/mL) was applied. After 24 h, the roots were challenged with *I. robusta* CBLJ-3 by inoculating 8 mm diameter plate plugs on the wounds. Control groups received sterile water instead of biocontrol agents. Disease progression was monitored by photographing lesion development at 5, 7, 9, and 11 days post-inoculation (dpi) and lesion diameter and depth were measured. Three biological replicates were conducted per treatment.

#### 2.3.3 Growth-promoting traits of biocontrol consortia

From the assays in vitro, 40 consortia demonstrating superior biocontrol efficacy were selected (Table S4). These were further evaluated for plant growth-promoting traits, which included nitrogen fixation using Ashby medium plates, potassium solubilization with Silicate bacteria medium and phosphorus solubilization by Pikovaskaia's medium. Transparent zone diameters around colonies were measured using the cross-streak method in 5 dpi. Consortium performance was compared to single-strain treatments to assess synergistic effects. Three replicates were conducted per treatment [13].

### 2.4 Inhibition of mycelia and spores on *I. robusta*

#### 2.4.1 Inhibition of mycelial growth on *I. robusta*

*I. robusta* CBLJ-3 was pre-cultured in potato dextrose broth (PDB) at 25 °C with shaking (150 rpm) for 48 h. Biocontrol treatments (Table S5) were introduced to the PDB cultures, while PDB without biocontrol agents served as the control. After 7 days of co-cultivation, mycelia were harvested by filtration through sterile gauze and rinsed three times with deionized water. Samples were dried at 80 °C to constant weight, and mycelial biomass was recorded. The mycelial growth inhibition rate was calculated as: Inhibition rate (%) = (control biomass − treatment biomass)/control biomass × 100​, Three biological replicates were performed per treatment [14].

#### 2.4.2 Inhibition of spore germination on *I. robusta*

A spore suspension of *I. robusta* CBLJ-3 was adjusted to 1 × 10^6^ spores/mL using deionized water. Equal volumes of spore suspension and biocontrol treatments were mixed and spread onto water agar plates. Control plates received spore suspension without biocontrol agents. Plates were inverted and incubated at 25 °C. Spore germination was assessed after 8 h using an optical microscope. The germination inhibition rate was calculated when control germination exceeded 90%: Inhibition rate (%) = (control germination rate − treatment germination rate)/control germination rate × 100 [14].

### 2.5 Antifungal spectrum of biocontrol consortia

The antimicrobial spectrum of biocontrol consortia was evaluated using the plate confrontation method. Eight pathogenic fungal strains (Table S1) were inoculated as 8 mm agar disks at the center of PDA plates. Biocontrol single strains or consortia (50 μL) were applied 27 mm radially from the fungal inoculum using a sterile punch. Plates were incubated at 25 °C, and inhibition zones were measured after 5 days [15].

### 2.6 Field experiment

Field trials were conducted in 2023 at the Baixi Base in Songjianghe Town, Fusong County, Jilin Province, an area acutely affected by *I. robusta*, where DNA concentrations of pathogen in soil reached 2031.64 fg/μL. The biocontrol consortia consisting of 10^6^ CFU/mL were applied through soil drenching, while the control plots were treated with sterile water. The experimental design involved randomizing the site into 2 m^2^ plots, with three of these plots selected for sampling. The planting arrangement included a row spacing of 20 cm and a plant spacing of 10 cm, resulting in approximately 60 plants per plot. Three-year-old ginseng plants with uniform size were planted at a depth of 7 cm and received consistent treatment. All ginseng roots were harvested at the maturity stage, and the severity of disease was assessed following the methodology described by Rahman and Punja [2].

### 2.7 Quantification of *I. robusta* via qPCR

*I. robusta* in ginseng roots and rhizosphere soil of different treatment was quantified using a lab-developed qPCR detection system [16]. DNA copy numbers were calculated from Ct values using a standard curve. Reactions (20 μL total volume) contained 2 × TransStart Tip Green qPCR SuperMix 10 μL, Forward/Reverse primers (10 μmol/L) 0.2 μL each, DNA template 2.0 μL, MgSO_4_ 2.0 μL, ddH_2_O to 20 μL. Thermocycling conditions: 95 °C for 10 min (initial denaturation), followed by 40 cycles of 95 °C for 10 s, 60 °C for 30 s.

### 2.8 Colonization evaluation of bacterial consortia in ginseng and rhizosphere soil

The antibiotic-labeled bacterial strains, including FG14-Rif^120^-Stre^250^ [17], NT35-Rif^160^-Stre^400^ [18], and NJ13-Rif^800^-Stre^2000^, were combined with an equal volume of bacterial suspension from unlabeled strains to create a mixed consortium with a final concentration of 1 × 10^6^ cfu/mL. Three-year-old healthy ginseng plants grown in the field were irrigated with the consortium at a concentration of 1 × 10^6^ cfu/mL (Table S6). Each ginseng plant was treated with a 100 mL infusion. During irrigation, the soil covering the ginseng was carefully removed to ensure that the bacterial solution thoroughly surrounded the roots. Sampling was conducted at 7, 14, 35, 49, and 63 dpi, with water irrigation serving as the control. Each experimental treatment was replicated three times.

The collected samples were disinfected using a sodium hypochlorite disinfection method. The disinfected samples were then separated into different parts: lateral roots, fibrous roots, epidermis, root core, stems, and leaves. Each part was weighed to obtain 2 g, which was then mixed with 20 mL of sterile water and ground in a mortar. After allowing the mixture to stand for 3 min, 100 μL of the supernatant was plated onto LB medium supplemented with antibiotics. The plates were incubated in a constant-temperature incubator, and the number of colonized colonies was recorded after regular observations. Additionally, fresh soil samples from the ginseng rhizosphere were periodically collected. A 2 g portion of each soil sample was transferred into a test tube containing 20 mL of sterile water and cultured for 30 min at 28 °C and 160 rpm. The cultured liquid was then serially diluted, and 100 μL of each dilution was plated onto antibiotic-containing LB medium to determine the number of colonized colonies [19].

### 2.9 Influence of biocontrol bacteria on enzymatic activity in ginseng rhizosphere soil

The soil samples analyzed in this study were collected from the designated field plot (section 2.2.8). The screened flora was combined by field experiment (Table S7), samples of rhizosphere soil from ginseng plants were obtained during three key growth periods: leaf spreading, fruiting and root expansion which was to assess the activity of four enzymes: urease [20], catalase, phosphatase [21] and sucrase [17].

### 2.10 Determination of induced defense enzyme gene expression in ginseng

Field-grown three-year-old healthy ginseng plants were root-irrigated with single or combined biocontrol strains (100 mL per plant). Control groups received sterile water. Tissues were sampled at 1, 3, 5, 7, 9, and 11 dpi, flash-frozen in liquid nitrogen, and stored at −80 °C. Three biological replicates were performed per treatment. Total RNA was isolated using RNAiso Plus and concentrations were quantified via NanoDrop 2000 spectrophotometry. RNA was reverse-transcribed into cDNA using M-MLV reverse transcriptase (TaKaRa). A qRT-PCR was performed on the fluorescent quantitative PCR instrument LC96 (Roche) with Fast SYBR Mixture under the following conditions: Initial denaturation: 95 °C for 10 min, 40 cycles: 95 °C for 10 s, 60 °C for 30 s. Reactions (20 μL) contained: 1 μL cDNA, 1 μL each forward/reverse primer (10 μmol/L), 10 μL 2 × Fast SYBR Mixture, and 7 μL ddH_2_O. Five defense enzyme genes (*phenylalanine ammonia lyase*, *β-1,3 glucanase*, *chitinase*, *superoxide dismutase*, *peroxidase*) were analyzed using *β-actin* as the internal reference. Primer sequences are listed in Table S8 (Shanghai Biotechnology Bioengineering Co., Ltd.). Relative gene expression was calculated using the 2^−△△Ct^ method [18,22].

### 2.11 Assessment of microbial interaction factor (MIF) in synergistic biocontrol bacteria

To evaluate microbial interactions, 15 mL centrifuge tubes containing PDA medium were prepared under two treatment protocols: treatment A: 0.4 mL biocontrol consortium, and treatment B: 0.4 mL biocontrol consortium + 0.4 mL *I. robusta* conidial suspension (final concentration of 1 × 10^7^ cfu/mL). Control tubes received 0.4 mL pathogen suspension with PDA medium. Tubes were incubated at 28 °C with shaking (180 rpm) for 48 h. Post-incubation, optical density (OD_600_) was measured using a microplate reader. Each treatment included six biological replicates and two technical replicates. The interaction strength was quantified as: MIF = log_10_(CPi/MPi), where CPi represents OD_600_ of the consortium-pathogen co-culture, and MPi was the mean OD_600_ of individual strains cultured separately. Interactions were classified as follows: MIF > 0 indicated a synergistic interaction, MIF = 0 meant a neutral interaction, and MIF < 0 showed an antagonistic interaction [23].

### 2.12 Microbial community profiling in ginseng rhizosphere soil

Rhizosphere soil samples were collected during three ginseng growth stages: leaf expansion**,** fruiting**,** and root expansion. A water-treated group (no bacteria) served as the control, with three biological replicates per treatment. Total DNA was extracted from 0.50 g soil using the CTAB method. The V3–V4 hypervariable regions of bacterial 16S rRNA were amplified with primers 341F (5′-CCTAYGGGRBGCASCAG-3′) and 806R (5′-GGACTACNNGGGTATCTAAT-3′), and the fungal ITS2 was amplified using primers ITS2F (5′-GCATCGATGAAGAACGCAGC-3′) and ITS2R (5′-TCCTCCGCTTATTGATATATGC-3′). Libraries were quantified on an Agilent 5400 system and sequenced on the Illumina NovaSeq 6000 platform (2 × 250 bp) [24]. Barcode and primer sequences were removed and reads were assembled into Raw Tags using FLASH (v2.2.7) [25]. Clean Tags were generated via QIIME2 (v2.9.1) [26] quality filtering and chimera removal. Sequences were clustered into Operational Taxonomic Units (OTUs) at 97% similarity using UPARSE (v7.0.1001) [27]. Taxonomic classification was assigned using the SILVA 138 SSU rRNA database [28]. The abundance and alpha diversity of OTUs were calculated. Spearman rank correlation analysis linked microbial taxa with soil enzyme activities (urease, catalase, phosphatase, sucrase) and ginseng growth metrics (root weight, chlorophyll content) were conducted. Heatmaps and histograms were visualized with the top 10 bacterial and fungal genera. The pairwise correlations and significance (p-values) were derived to identify microbial taxa associated with plant health or pathogen suppression.

### 2.13 Statistical analysis

All the results were statistically analyzed using one-way analysis of variance (ANOVA) and the least significant difference (LSD) test, with a significance level set at 0.05. Data presented in all figures represent the mean ± standard deviation (SD) of three replicates. The statistical analyses were performed with SPSS 26.0 [17].

## 3 Results

### 3.1 Construction of biocontrol consortia

The compatibility among 10 candidate biocontrol strains demonstrated no antagonism between the following strain groups, Group 1: FG14, NJ13, JJ5-2, TU26, JI6, JA26; Group 2: NJ13, JI39-2, TJ23-2, TU26, JI6, JJ5-2; Group 3: NJ13, JA38, TU26, JI6, JJ5-2. These compatible strains were selected to assemble consortia (Table 1). Strains were combined at equal ratios (1:1) to construct consortia with increasing strain richness (1–6 strains per consortium), following a full factorial combinatorial design (Table 1). A total of 98 distinct consortia were prepared to evaluate diversity-dependent effects on biocontrol efficacy.

**Table 1:** Determination of the antagonistic effect between the biocontrol strains (mm).

Strains	FG14	NT35	NJ13	TJ23-2	JJ5-2	JA38	TU26	JI6	JI39-2	JA26
FG14	-	-	-	-	-	6	-	-	6	-
NT35	-	-	-	-	-	8	-	-	6	-
NJ13	-	-	-	-	-	-	-	-	-	-
TJ23-2	-	-	-	-	-	10	-	-	-	-
JJ5-2	-	-	-	-	-	-	-	-	-	-
JA38	6	8	-	10	-	-	-	-	-	-
TU26	-	-	-	-	-	-	-	-	-	-
JJ6	-	-	-	-	-	-	-	-	-	-
JI39-2	6	6	-	-	-	-	-	-	-	-
JA26	-	-	-	-	-	-	-	-	-	-

Note: the number in the table represents the size of the antagonistic inhibition zone between the strains.

- represents no antagonism between the strains which were good compatibility.

### 3.2 Evaluation of biocontrol and growth-promoting effects of synergistic consortia

#### 3.2.1 Inhibitory effects of biocontrol consortia

**Fig. 1:**
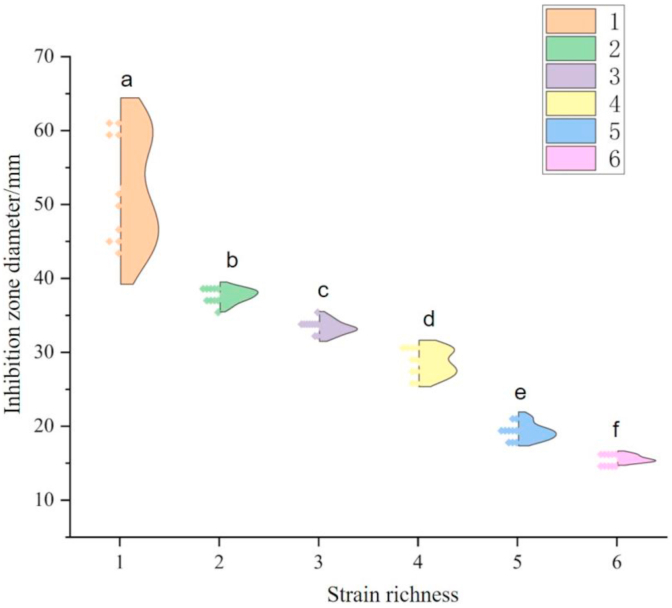
Inhibitory effect of different strain combinations on pathogen of *Ilyonectria robusta.* Note: Different colors were different richness combinations. The dots in the figure represent the inhibition diameter of different combinations against *Ilyonectria robusta*.

**Fig. 2:**
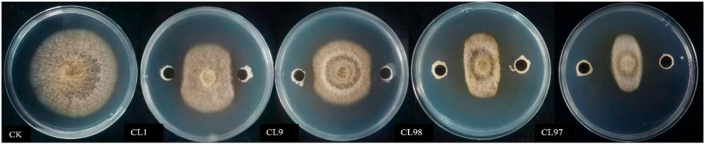
Inhibitory effect of different biocontrol bacteria combinations on *Ilyonectria robusta*.

There were significant differences of inhibition on mycelial growth of *I. robusta* CBLJ-3 between single-strain treatments and multi-strain consortia, with consortia demonstrating superior efficacy (Fig. 1). Among the single-strain treatments, CL1 demonstrated the highest inhibition efficacy, achieving a 28% inhibition rate. In contrast, the six-strain consortia CL98 and CL97 exhibited significantly enhanced performance, with inhibition rates of 75% and 74%, respectively, representing 167% and 164% improvements over CL1. Similarly, the five-strain consortia CL95 and CL94 showed substantial inhibition rates of 73%, which were 160% higher than that of CL1. The inhibition efficacy was positively correlated with consortium richness, as the six-strain consortia displayed the most potent suppression of pathogen growth, which was further corroborated by the reduced mycelial diameters (Fig. 2). These findings highlight the significant potential of microbial consortia in improving biocontrol outcomes and provide a robust foundation for future field applications.

#### 3.2.2 In vitro evaluation of biocontrol strains/consortia against *I. robusta* in ginseng roots

**Fig. 3:**
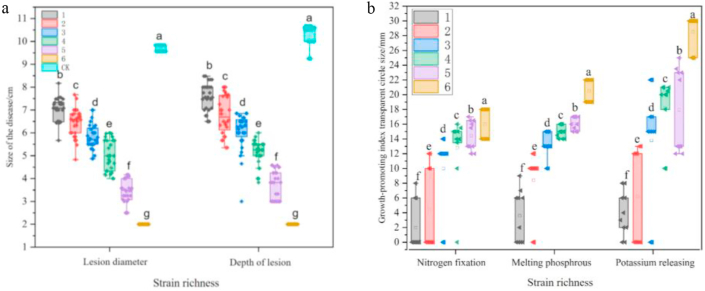
In vitro effect of control and growth-promoting activities of by biocontrol consortia. a. The depth and surface diameter of disease lesion in ginseng root by different consortia treatment. b. The diameter of the transparent circle through the determination of nitrogen-fixing, melting phosphrous and potassium releasing. Note: Different colors are different richness flora combinations. The dots in the figure represent the antibacterial diameter of different flora combinations against ginseng rust rot pathogens.

Ginseng roots were treated with biocontrol consortia (Table S3) and subsequently inoculated with *I. robusta*. The control group characterized by extensive lesions, deep infection and severe root rot, while biocontrol group with smaller lesions, lighter discoloration, and reduced infection depth compared to the control. Significant differences in preventive effect were observed between single-strain and consortium treatments (Fig. 3). Lesion diameter and depth decreased significantly with increasing consortium richness. No severe infection symptoms were observed in consortia-treated groups. From 98 biocontrol consortium combinations, 40 were identified as highly effective in preventing infection of *I. robusta* (Table S4).

#### 3.2.3 In vitro growth-promoting activities by biocontrol consortia

All the 40 selected biocontrol consortia exhibited potassium-solubilizing activity, 30 demonstrated nitrogen-fixing capability and 31 solubilized inorganic phosphorus (Fig. 3). Consortia outperformed single-strain treatments in all three growth-promoting activities. Increased consortium richness correlated with enhanced growth-promoting effects. Six-strain consortia produced the largest transparent zones, indicating superior nutrient solubilization. From the initial 40 consortia, 29 were identified as highly effective in promoting plant growth and preventing disease (Table S5). These results highlight the synergistic potential of multi-strain consortia in enhancing ginseng growth and soil nutrient availability.

#### 3.2.4 Effects of biocontrol consortia spores of *I. robusta* CBLJ-3

The inhibitory effects of consortia on the mycelium and spores production of *I. robusta* CBLJ-3 increased with consortium richness (Table S5). Six- and five-strain consortia exhibited the strongest suppression, with mycelial dry weight inhibition rates 130% and 120% higher than single-strain treatments, respectively. Four- and three-strain consortia showed moderate inhibition, with rates 90% and 70% higher than single-strain treatments, and two-strain consortia demonstrated the lowest inhibition, with rates 60% higher than single-strain treatments. Spore production inhibition followed a similar trend, with six-strain consortia achieving 40% higher suppression compared to single-strain treatments (Fig. S1).

#### 3.2.5 Antifungal spectrum of biocontrol consortia

**Fig. 4:**
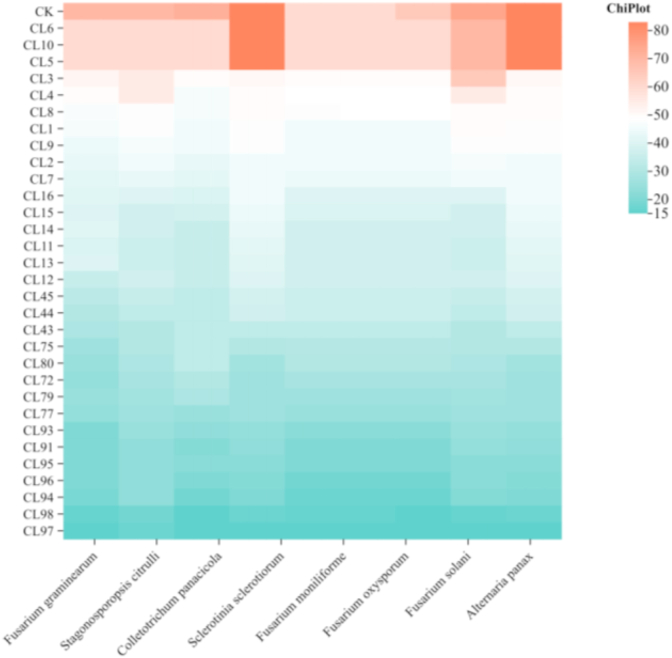
Antifungal spectrum of biocontrol bacteria group.

The consortia exhibited significant inhibition against all eight pathogenic fungi associated with plant diseases, demonstrating broad-spectrum antifungal potential. Inhibition efficacy varied among consortia, with multi-strain formulations outperforming single-strain treatments (Fig. 4). Consortia achieved 150% to 200% higher inhibition rates compared to single-strain treatments. Antifungal activity increased with consortium richness. Six-strain consortia CL97 showed the strongest inhibition, with the widest inhibition zones, indicating maximal suppression of pathogen growth (Fig. S2).

### 3.3 Field biocontrol efficacy and quantitative change of *I. robusta*

The field trial results demonstrated that microbial consortia significantly reduced the disease index of ginseng rust rot compared to both single-strain treatments and the water control. Six consortia exhibited control efficacy exceeding 50%, with CL95 showing the highest efficacy at 66.67%, followed closely by CL92 at 62.96%. Other effective consortia included CL94, CL79, CL80, and CL44, with control efficacies of 59.26%, 59.26%, 55.56%, and 51.85%, respectively. Quantitative analysis of *I. robusta* in ginseng tissues (Table 2) revealed that the biocontrol bacteria effectively reduced the pathogen load in host plants. Among single-strain treatments, CL1 demonstrated the lowest pathogen content in both ginseng tissues (112.59 fg/μL) and soil (71.73 fg/μL). The efficacy of pathogen suppression was positively correlated with strain diversity, with five-strain consortia showing optimal performance in controlling both mycelial growth and spore production of pathogens. Specifically, CL95, CL92, and CL94 treatments reduced *I. robusta* content by 34.0–47.0% and 72.6–75.0% compared to NJ13. Four-strain consortia (CL79, CL69, and CL44) showed intermediate efficacy, reducing pathogen content by 8.2–32.6% and 17.8–72.1% relative to NJ13.

**Table 2:** qPCR detection of *I. robusta* in ginseng roots and soil treated with biocontrol bacteria in the field.

Combinations	(fg/μL)	(fg/μL)	Combinations	(fg/μL)	(fg/μL)
CL1	112.59 ± 1.93 c	71.73±±1.70 c	CL44	103.28 ± 1.35 c	58.97 ± 1.73 c
CL2	528.74 ± 2.53 kl	573.55 ± 3.67 kl	CL69	87.10 ± 0.43 bc	33.06 ± 0.14 bc
CL3	325.42 ± 2.88 h	350.20 ± 2.08 h	CL75	138.80 ± 1.45 d	105.60 ± 0.66 d
CL4	507.51 ± 3.45 k	530.70 ± 2.25 k	CL76	164.23 ± 1.68 f	115.30 ± 1.14 f
CL5	302.41 ± 2.64 g	267.95 ± 1.41 g	CL79	75.87 ± 0.58 b	20.53 ± 0.63 b
CL6	550.87 ± 2.34 l	570.30 ± 3.35 l	CL80	152.29 ± 1.08 e	145.80 ± 1.73 e
CL7	146.17 ± 1.24 e	121.20 ± 1.02 e	CL91	127.05 ± 1.74 d	87.55 ± 0.13 d
CL8	475.71 ± 2.25 j	500.88 ± 3.22 j	CL92	74.25 ± 1.18 b	19.64 ± 0.31 b
CL9	297.88 ± 1.44 g	206.78 ± 1.09 g	CL93	295.32 ± 1.32 g	200.55 ± 1.67 g
CL10	353.99 ± 1.36 i	384.30 ± 3.73 i	CL94	78.70 ± 0.87 b	21.92 ± 0.42 b
CL11	109.48 ± 1.11 c	69.85 ± 0.23 c	CL95	59.71 ± 0.74 a	17.17 ± 0.16 a
CL12	117.81 ± 1.05 c	72.33 ± 1.21 c	CL96	130.38 ± 1.31 d	54.58 ± 0.31 d
CL13	123.27 ± 1.28 d	83.64 ± 1.42 d	CL97	162.82 ± 1.46 f	160.46 ± 1.44 f
CL14	333.96 ± 3.04 h	340.86 ± 2.63 hl	CL98	167.45 ± 1.77 f	163.33 ± 1.62 f
CL43	145.86 ± 1.20 e	126.02 ± 1.17 e	CK	618.92 ± 5.10 m	764.78 ± 6.98 m

**Fig. 5:**
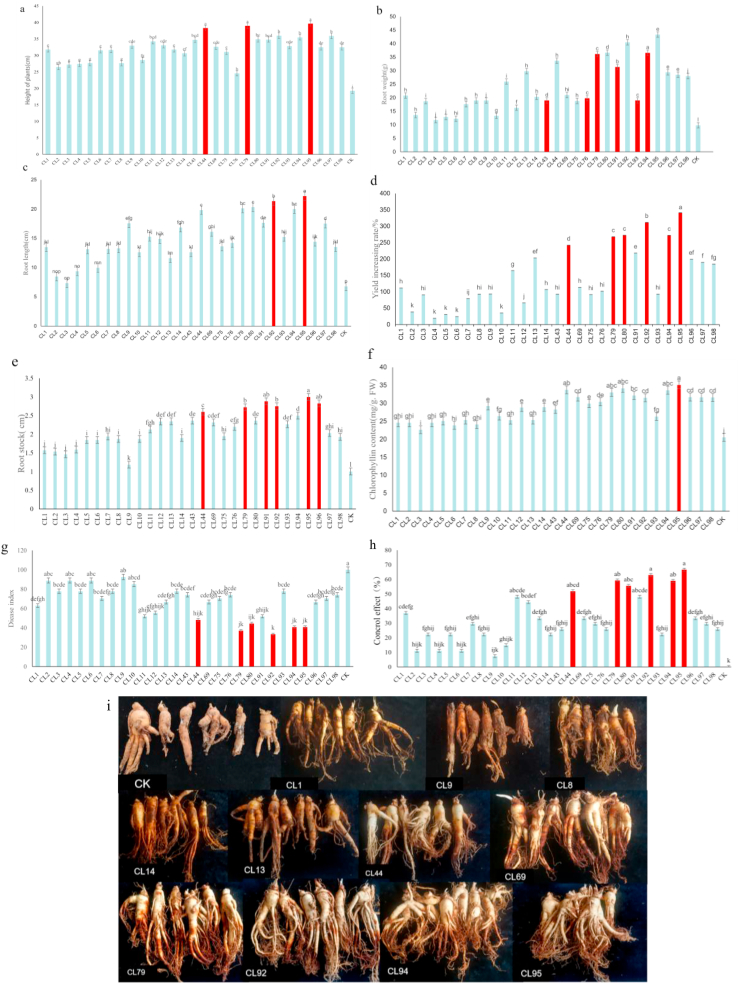
Effects of single biocontrol strain and biocontrol flora on the growth of ginseng in the field. The effects of biocontrol bacteria on ginseng plant height (a), root fresh weight (b), root length (c), growth rate (d), main root diameter (e), chlorophyll content (f), disease index (g) and control effect (h) were determined. The results showed that the effects of biocontrol bacteria on ginseng main root plant height (a), root fresh weight (b), root length (c), growth rate (d), main root diameter (e), chlorophyll content (f), disease index (g) and control effect (h, i) were significantly better than those of biocontrol single strain treatment. Means of significant values are separated by Fisher's Least Significant Difference test (LSD) (p = 0.05), and denoted by lowercase letters.

The consortia treatments significantly enhanced ginseng growth parameters compared to controls. Root length in consortia-treated plants was 250% greater than the water control and 37%–229% higher than single-strain treatments (Fig. 5a). Similarly, fresh root weight showed a 340% increase over the water control and 108% to 217% improvement compared to single-strain treatments (Fig. 5b). The root diameter measurement showed that the root diameter of (Fig. 5c and e) increased by 198 % compared with the water control, and increased by 54%–154% compared with the single strain treatment. The chlorophyll content increased by 79% compared with the water control, and increased by 20%–55% compared with the single strain treatment (Fig. 5f). From the 29 consortia evaluated in field trials, six high-performing combinations (CL95, CL92, CL94, CL79, CL80, and CL44) were identified for further mechanistic studies (Table S6). These selected consortia demonstrated dual functionality, effectively suppressing rusty root rot while simultaneously promoting ginseng growth, highlighting their potential for integrated disease management and yield enhancement strategies.

### 3.4 Colonization dynamics in ginseng and rhizosphere soil

The double antibiotic-labeled strains successfully established colonization in both ginseng tissues and rhizosphere soil over a 63-day period. Comparative analysis revealed that microbial consortia demonstrated superior colonization capabilities across all ginseng tissues compared to single-strain treatments. In the rhizosphere soil, colonization peaked at 7 dpi and subsequently showed a steady decline (Fig. S3g). Tissue-specific colonization patterns revealed distinct spatial and temporal dynamics. The highest colonization in the root epidermis was observed at 7 dpi, exceeding levels detected in fibrous roots, lateral roots, and root cores (Fig. S3c). Notably, colonization in root cores and lateral roots exhibited a progressive increase from 14 to 35 dpi, surpassing epidermal and fibrous root colonization by 35 dpi. Between 49 and 63 dpi, a significant shift in colonization patterns was observed, with stems and leaves showing higher colonization levels than root tissues, indicating upward migration of labeled strains from the rhizosphere. All tissues exhibited a sharp decline in colonization rates after 49 dpi, mirroring the trend observed in rhizosphere soil. For example, the biocontrol bacteria CL95 showed excellent colonization efficiency, which was significantly better than single strain treatment: 50%–275% higher in leaves, 43%−400% higher in stems, 215%–400% higher in epidermis, 110%−330% higher in root core, 33% –280% higher in lateral roots, 33%–100% higher in fibrous roots, and 100%–200% higher in rhizosphere soil.

Based on the observed colonization dynamics, we propose a spatio-temporal trajectory of biocontrol strain colonization: initial establishment in rhizosphere soil, followed by colonization of root epidermis and fibrous roots, subsequent penetration into root cores, and final translocation to stems and leaves. This systematic colonization pattern underscores the superior ability of consortia, particularly CL95, to establish and maintain presence throughout the ginseng plant system, demonstrating their potential for effective biocontrol applications.

### 3.5 Effects of biocontrol consortia on soil enzyme activity and defense enzyme gene expression of ginseng

**Fig. 6:**
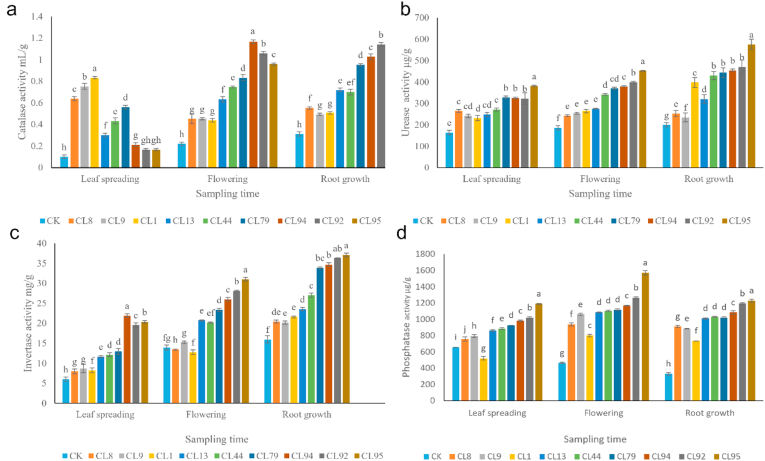
Effects of single biocontrol strain and flora on four soil enzyme activities at different stages. (a) catalase (b) urease (c) invertase (d) phosphatase.

The application of both biocontrol consortia and single-strain treatments significantly enhanced enzymatic activities in ginseng rhizosphere soil over a 90-day experimental period. Four key soil enzymes of urease, catalase, sucrase, and phosphatase showed consistent activity increases throughout the study. Comparative analysis revealed that consortia-treated soils consistently outperformed both single-strain treatments and the water control in enzymatic activity enhancement. Notably, the biocontrol consortium CL95 demonstrated exceptional performance during the root expansion period, significantly boosting soil enzyme activities and indicating substantial improvements in nutrient cycling and soil health. Compared with the single strain CL1, the soil phosphatase activity of CL95 treatment increased by 100%, the sucrase activity increased by 140%, the catalase activity increased by 180%, and the urease activity increased by 150%. Enzyme activities reached their maximum levels on the 90th day, demonstrating the long-term stability and sustained effectiveness of consortia treatments (Fig. 6).

**Fig. 7:**
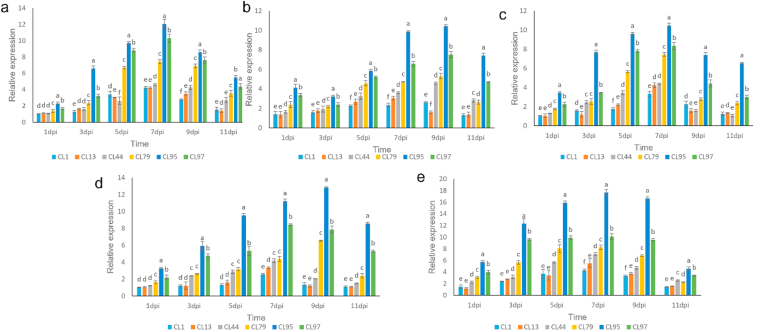
Changes in the relative expression of five ginseng defense enzyme genes induced by biocontrol single strains and flora. (a) *β-1,3 glucanase* (b) *phenylalanine ammonia lyase* (c) *peroxidase* (d) *superoxide dismutase* (e) *chitinase*.

In parallel, both consortia and single-strain treatments upregulated the expression of five key defense enzyme synthesis genes in ginseng plants (Fig. 7). The gene expression patterns followed a characteristic trend, showing an initial increase followed by a gradual decline. Among all treatments, the mixed strain CL95 showed the most obvious effect, and the induced gene expression level was 75%–167% higher than that of the single strain NJ13 (CL1). This study further confirmed that multi-strain formulations were significantly more effective in activating defense enzyme genes compared to single-strain treatments, highlighting the synergistic benefits of microbial consortia in plant defense mechanisms.

### 3.6 Analysis of MIF in biocontrol consortia

**Fig. 8:**
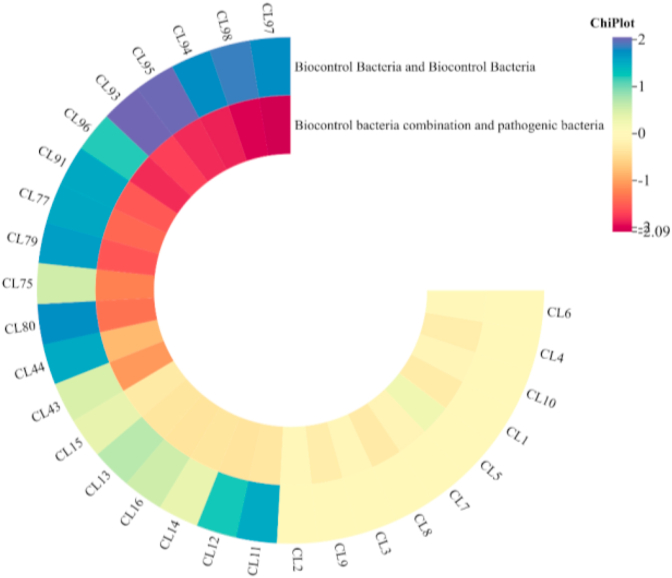
MIF interaction analysis of biotic flora.

The interaction patterns among biocontrol consortia and between consortia and pathogens were analyzed using the MIF. Among consortia, there were 20 of 30 combinations exhibited synergistic interactions (Fig. 8). The average promotion effect increased with strain richness. The top five synergistic consortia were CL95, CL92, CL93, CL98, and CL97, all comprising 5–6 strains. The 28 of 30 combinations showed antagonistic interactions with *I. robusta* CBLJ-3. The strongest antagonistic effects were observed in CL97, CL98, CL93, CL95, and CL96. Antagonistic strength against pathogens increased with consortium richness, highlighting the diversity effect in microbial interactions. The MIF analysis revealed that multi-strain consortia predominantly exhibit synergistic interactions among themselves and antagonistic interactions with pathogens. High-richness consortia demonstrated superior pathogen suppression, further emphasizing the importance of strain diversity in biocontrol efficacy.

### 3.7 Effects of biocontrol consortia on rhizosphere microbial communities

#### 3.7.1 Microbial community diversity in ginseng rhizosphere soil

The α-diversity indices (Chao1 and Shannon) were used to assess the richness and diversity of bacterial and fungal communities in ginseng rhizosphere soil (Fig. S4). In bacterial communities, consortia-treated soils exhibited higher bacterial richness (Chao1 index) compared to single-strain treatments across all the three growth periods. The consortia, particularly CL95 and CL92, significantly enhanced bacterial diversity (Shannon index). In fungal communities, the richness initially decreased but stabilized over time, with consortia treatments outperforming single-strain treatments. The diversity showed a U-shaped trend, with consortia treatments maintaining higher diversity than single-strain treatments.

#### 3.7.2 Dynamics of bacterial and fungal communities across ginseng growth periods

The top 10 bacterial and fungal genera were analyzed to assess community composition changes (Fig. S5). The consortia increased the diversity and abundance of beneficial bacteria in ginseng rhizosphere soil while reducing the abundance of pathogenic fungi. Beneficial Bacterial genera such as *Candidatus* Udaeobacter, *Candidatus* Nitrosotalea, *Sphingomonas*, *Achromobacter*, *Sphingobium*, and *Stenotrophomonas* increased in abundance over time in consortia-treated soils. These shifts in microbial composition improved soil health and indirectly suppressed pathogen activity. In fungal communities, *Ilyonectria* genus abundance was significantly reduced in consortia-treated soils, particularly during the flowering and root expansion stages of ginseng. CL95 and CL92 were most effective in reducing *Ilyonectria* levels, demonstrating their potential for disease control. The treatment of CL92 and CL95 effectively reduced the content of harmful bacteria *Ilyonectria* and *Plectosphaerella* in the soil, promoted the increase of beneficial bacteria *Umbelopsis*, *Trichoderma* and *Pseudogymnoascus* in the soil, and played a key role in the prevention and treatment of diseases.

#### 3.7.3 Correlation analysis between microbial communities and functional traits

Spearman correlation analysis was performed to evaluate relationships among microbial communities, soil enzyme activities, disease index and ginseng growth (Fig. S6). In bacterial communities, the disease index and four enzyme activities were positively correlated with *Rhodanobacter*, *unidentified-Gemmatimonadaceae* and *unidentified-Chitinophagaceae*, and negatively correlated with *Achromobacter*. After CL95 treatment, the disease index was significantly positively correlated with *Rhodanobacter* and negatively correlated with *Sphingomonas*, *Stenotrophomonas*, *Sphingobacterium* and *Pedobacter*. The use of flora promotes the increase of beneficial bacteria and inhibits harmful bacteria. Root weight and enzyme activities were negatively correlated with *Rhodanobacter*, *unidentified-Gemmatimonadaceae*, and *unidentified-Chitinophagaceae*, but positively correlated with *Achromobacter* in the single-strain treatments. Root weight was negatively correlated with *Rhodanobacter* and positively correlated with *Sphingomonas*, *Stenotrophomonas*, *Sphingobacterium*, and *Pedobacter* in the CL95 treatment.

In fungal communities of single-strain treatments, the disease index and four enzyme activities were positively correlated with *Ilyonectria*, *Plectosphaerella* and *Solicoccozyma*, and negatively correlated with *Penicillium*, *Vishniacozyma*, *Fusarium* and *Trichoderma*. After CL95 treatment, the disease index was significantly positively correlated with *Ilyonectria*, *Plectosphaerella*, *Leptodontidium*, *Clonostachys*, *Penicillium*, and significantly negatively correlated with *Mortierella* and *Pseudogymnoascus*. Root weight and enzyme activities were negatively correlated with *Ilyonectria*, *Plectosphaerella*, and *Solicoccozyma*, but positively correlated with *Penicillium*, *Vishniacozyma*, *Fusarium*, and *Trichoderma*. In the CL95 treatment, root weight was also negatively correlated with *Ilyonectria*, *Plectosphaerella*, *Leptodontidium*, *Clonostachys*, and *Penicillium*, but positively correlated with *Mortierella* and *Pseudogymnoascus*.

## 4 Discussion

### 4.1 Construction of synthetic microbial communities

Functional bacteria are microorganisms that perform specific functions, either in the same or different forms [29]. A synthetic microbial community (SynCom) is an artificially created consortium of two or more microbial species cultured under controlled conditions [30]. SynComs have been applied in the biocontrol of plant diseases caused by fungi, oomycetes, bacteria, viruses, and nematodes, often outperforming single-strain applications [31]. A number of studies have confirmed that multi-strain combinations are more prominent in inhibiting soil-borne diseases, promoting plant growth, and improving stress resistance. The simplified high-efficiency flora can still maintain the biocontrol effect similar to the complex flora, and the colonization ability is stronger. Synthetic microbial communities can also improve soil microecology and repair degraded environments, showing good application potential [[32], [33], [34], [35], [36], [37], [38], [39]]. In this study, we isolated bacteria from both diseased and healthy ginseng roots, suggesting that the root-associated microbial community has potential for controlling soil-borne diseases. We isolated multiple functional bacteria from the rhizosphere of ginseng and constructed 98 biocontrol bacteria combinations. The screened high-efficiency bacteria such as CL95 and CL92 were significantly better than single strains in terms of antagonistic effect, in vitro control effect and growth promotion effect. With the increase of strain richness, the disease inhibition effect continued to increase.

While not all enriched microorganisms are directly involved in disease resistance, some, like *Arthrobacter nicotinovorans* JI39, promote ginseng growth and could serve as microbial fertilizers [40]. Another strain, *Pseudomonas thivervalensis* JI6, demonstrated growth-promoting effects, rhizosphere colonization, upregulation of defense enzyme genes, and modulation of soil microbial communities, ultimately enhancing ginseng yield and saponin content [13]. Some SynComs were constructed for ginseng rust rot control, selecting bacteria based on their antagonistic properties and growth-promoting. Laboratory and field tests confirmed that the SynCom outperformed individual strains in disease suppression (Fig. 1, Fig. 2). The SynComs exhibited superior growth-promoting effects, likely due to synergistic interactions among its members (Fig. 3). Additionally, the SynComs demonstrated broad-spectrum antimicrobial activity, surpassing that of individual strains (Fig. 4). These findings underscore the importance of understanding microbial interactions for developing effective biocontrol agents.

Field trials revealed that the SynCom not only controlled rust rot more effectively than single strains but also promoted ginseng growth (Fig. 5). However, the control efficacy did not always correlate with microbial richness, as a five-strain SynCom outperformed a six-strain one. This discrepancy may stem from competition, environmental factors, or the presence of non-functional bacteria in the SynCom. Further mechanistic studies are needed to optimize SynCom composition and functionality.

### 4.2 Mechanisms of synergistic effects in biocontrol SynComs

The enhanced disease control and growth promotion by SynComs can be attributed to several mechanisms:

**Niche Differentiation**: Members of the SynCom occupy distinct ecological niches, reducing competition and enhancing stable colonization in the host plant and soil [41].

**Interspecies Interactions**: Microbial interactions within the SynCom can upregulate disease-related genes, enhancing plant defense mechanisms [4].

**Soil Enzyme Activity Modulation**: SynComs can alter soil enzyme activities, influencing microbial community structure and diversity [42].

**Microbial Community Recruitment**: SynComs can recruit beneficial microorganisms, improving the rhizosphere environment and suppressing pathogens [43].

**Induced Systemic Resistance (ISR)**: SynComs can activate plant immune responses, providing broad-spectrum disease resistance [44].

#### 4.2.1 Enhanced microbial colonization

Effective colonization of the rhizosphere is crucial for biocontrol efficacy. Dong et al. reported that SynComs reduced pathogenic *Fusarium* populations in rhizosphere soil, thereby limiting pathogen colonization [45]. In this study, the SynCom enhanced the colonization of beneficial bacteria in the ginseng rhizosphere, likely through synergistic interactions that promoted biofilm formation and microbial migration (Fig. S3). SynComs can also degrade complex compounds that individual strains cannot, further enhancing microbial growth and colonization [46]. For example, *Acinetobacter* sp. Y2 and *Scedosporium* sp. ZYY produced biosurfactants that reduced crude oil toxicity, facilitating ZYY growth [47]. These interactions may reduce the pathogenicity of rust rot pathogens in soil, highlighting the importance of microbial synergy in effective soil colonization.

#### 4.2.2 Induction of plant systemic resistance

Plants have evolved defense mechanisms, including the synthesis of defense enzymes, to protect against pathogens. SynComs can activate these defenses, enhancing plant resistance. In this study, the SynCom upregulated the expression of defense enzyme genes, such as chitinase and oxidase, in ginseng (Fig. 7). Enzymes like CAT, SOD, POD, and PAL play critical roles in plant defense, with PAL promoting phenol production and lignification to block pathogen invasion [[48], [49], [50]]. *Bacillus* species, known for forming biofilms around roots, can accelerate plant growth and induce systemic resistance [51,52]. Similarly, strain JI6 enhanced ginseng's resistance to rust rot by upregulating defense enzyme activity [13]. The SynCom also improved ginseng's damage repair capacity, increasing chlorophyll content, photosynthesis, and defense enzyme activity, thereby enhancing tolerance to biotic stress.

#### 4.2.3 Microbial community analysis

Soil microbial interactions are vital for ecosystem functions, including nutrient cycling and plant growth support [17]. High-throughput sequencing revealed that the SynCom altered the ginseng rhizosphere microbiome, increasing microbial diversity and network complexity (Fig. S4, Fig. S5). The SynCom enriched beneficial bacteria, such as *Sphingomonas* and *Stenotrophomonas*, which, although not directly antagonistic to *Ilyonectria robusta*, activated ISR-related enzymes, indicating indirect pathogen suppression through signaling pathways. Spearman analysis showed that the SynCom enriched beneficial bacteria positively correlated with ginseng root weight, suggesting a role in growth promotion (Fig. S6). These findings highlight the potential of SynComs to modulate soil microbiomes for disease control and plant health.

#### 4.2.4 Impact on soil enzyme activity

Soil enzymes are critical indicators of soil health and microbial activity. The SynCom increased the activity of urease, catalase, and alkaline phosphatase in ginseng rhizosphere soil, particularly during the root enlargement stage (Fig. 6). These enzymes are involved in nutrient cycling, such as nitrogen and phosphorus mineralization, which are essential for plant growth. For example, *Mortierella* species, enriched in SynCom-treated soil, can solubilize phosphorus, enhancing its availability to plants [53]. The SynCom also increased β-glucosidase activity, which is involved in organic matter decomposition and nutrient release. These changes in enzyme activity suggest that the SynCom improves soil health, thereby enhancing ginseng growth and disease resistance.

### 4.3 Future perspectives

The findings of this study provide a foundation for the application of SynComs in ginseng cultivation. Future research should focus on:

**Optimizing SynCom Composition**: Identifying the key functional strains within SynComs and their interactions to enhance disease control and growth promotion.

**Mechanistic Studies**: Elucidating the molecular mechanisms underlying SynCom-induced plant resistance and microbial colonization.

**Field Applications**: Scaling up SynCom applications in field conditions, considering environmental factors and soil microbiota dynamics.

**Sustainable Agriculture**: Integrating SynComs into sustainable agricultural practices to reduce reliance on chemical pesticides and fertilizers.

In conclusion, this study demonstrates the potential of SynComs for ginseng rust rot control and growth promotion. By leveraging microbial interactions, SynComs offer a promising strategy for sustainable agriculture, enhancing plant health and soil ecosystem functionality.

## CRediT authorship contribution statement

**Meng Tian:** Writing – original draft, Visualization, Investigation. **Yu Wang:** Investigation. **Keying Sun:** Investigation. **Lingxin Kong:** Investigation. **Xiaolin Chen:** Visualization, Conceptualization. **Kaixin Zhang:** Visualization, Conceptualization. **Lina Yang:** Investigation. **Baohui Lu:** Investigation. **Jie Gao:** Visualization, Conceptualization. **Wanpeng Hou:** Investigation. **Yongzhe An:** Investigation. **Yun Jiang:** Writing – review & editing, Supervision, Funding acquisition. **Changqing Chen:** Writing – review & editing, Supervision, Funding acquisition.

## Declaration of competing interest

The authors declare the following financial interests/personal relationships which may be considered as potential competing interests: Wanpeng Hou and Yongzhe An are currently employed by Jilin Shenwang Plant Protection Co., Ltd.

## References

[1] Zhou Y.Q., Wang H.K., Sun J.X., Wicaksono W.A., Liu C., He Y.H. (2025). Phenazines contribute to microbiome dynamics by targeting topoisomerase IV. Nat Microbiol.

[2] Rahman M., Punja Z.K. (2005). Factors influencing development of root rot on ginseng caused by *Cylindrocarpon destructans*. Phytopathology.

[3] Macia J., Manzoni R., Conde N., Urrios A., de Nadal E., Solé R. (2016). Implementation of complex biological logic circuits using spatially distributed multicellular consortia. PLoS Comput Biol.

[4] Li Z.F., Bai X.L., Jiao S., Li Y.M., Li P.R., Yang Y. (2021). A simplified synthetic community rescues *Astragalus mongholicus* from root rot disease by activating plant-induced systemic resistance. Microbiome.

[5] Veerubommu S., Nandina K. (2011). Biological management of vascular wilt of tomato caused by *Fusarium oxysporum* f.sp. *lycospersici* by plant growth-promoting rhizobacterial mixture. Biol Control.

[6] Zhang L.N., Wang D.C., Hu Q., Dai X.Q., Xie Y.S., Li Q. (2019). Consortium of plant growth-promoting rhizobacteria strains suppresses sweet pepper disease by altering the rhizosphere microbiota. Front Microbiol.

[7] Santhanam R., Menezes R.C., Grabe V., Li D.P., Baldwin L.T., Groten K. (2019). A suite of complementary biocontrol traits allows a native consortium of root-associated bacteria to protect their host plant from a fungal sudden-wilt disease. Mol Ecol.

[8] Gonin M., SalasG I., Gopaulchan D., Frene J.P., Roden S., Van de Poel B. (2023). Plant microbiota controls an alternative root branching regulatory mechanism in plants. Proc Natl Acad Sci U S A.

[9] Emmenegger B., Massoni J., Pestalozzi C.M., Bortfeld M.M., Maier B.A., Vorholt J.A. (2023). Identifying microbiota community patterns important for plant protection using synthetic communities and machine learning. Nat Commun.

[10] Qiao Y.Z., Wang Z.D., Sun H., Guo H.Y., Song Y., Zhang H. (2024). Synthetic community derived from grafted watermelon rhizosphere provides protection for ungrafted watermelon against *Fusarium oxysporum* via microbial synergistic effects. Microbiome.

[11] Jiang G.F., Zhang Y.L., Gan G.Y., Li W.L., Wan W., J Y.Q. (2022). Exploring rhizo-microbiome transplants as a tool for protective plant-microbiome manipulation. ISME Commun.

[12] Jiang G.F., Zhang Y.L., Chen M., Ramoneda J., Han L.L., Shi Y. (2024). Effects of plant tissue permeability on invasion and population bottlenecks of a phytopathogen. Nat Commun.

[13] Liu T.T., Zhang J.Y., Wang T., Li Z.Y., Liang H.J., Jiang C.Y. (2024). The novel *Pseudomonas thivervalensis* strain JI6 promotes growth and controls rusty root rot disease in Panax ginseng. Biol Control.

[14] Li X., Li M.T., Liu X.K., Jiang Y.L., Zhao D.F., Gao J. (2022). RNA-Seq provides insights into the mechanisms underlying *Ilyonectria robusta* responding to secondary metabolites of *Bacillus methylotrophicus* NJ13. J Fungi.

[15] Xu S.D., Liu Y.X., Cernava T., Wang H.K., Zhou Y.Q., Xia T. (2022). Fusarium fruiting body microbiome member *Pantoea agglomerans* inhibits fungal pathogenesis by targeting lipid rafts. Nat Microbiol.

[16] Jiang Y.L., Kong L.X., Jin H., Yan D., Li X., Zhang Y.L. (2022). Development of a novel real-time quantitative PCR method for detection of *Ilyonectria robusta*, the predominant species causing ginseng rusty root rot. Plant Dis.

[17] Wang J., Wang J.R., Liu T.T., Li X., Gao J., Jiang Y. (2023). *Bacillus amyloliquefaciens* FG14 as a potential biocontrol strain against rusty root rot of *Panax ginseng*, and its impact on the rhizosphere microbial community. Biol Control.

[18] Li X.Q., Wang J.R., Shen H., Xing C.X., Kong L.X., Song Y. (2024). Biocontrol and growth promotion potential of *Bacillus velezensis* NT35 on *Panax ginseng* based on the multifunctional effect. Front Microbiol.

[19] Liu Y.P., Shu X., Chen L., Zhang H.H., Feng H.C., Sun X.T. (2023). Plant commensal type VII secretion system causes iron leakage from roots to promote colonization. Nat Microbiol.

[20] Zhao R., Liu J.Y., Xu N., He T.Y., Meng J., Liu Z.Q. (2022). Urea hydrolysis in different farmland soils as affected by long term biochar application. Front Environ Sci.

[21] Wang L., Yang F., E Y.Y., Yuan J., Raza W., Huang Q.W. (2016). Long-term application of bioorganic fertilizers improved soil biochemical properties and microbial communities of an apple orchard soil. Front Microbiol.

[22] Chen Z.W., Halford N.G., Liu C.H. (2023). Real-time quantitative PCR: primer design, reference gene selection, calculations and statistics. Metabolites.

[23] Lan J.R., Huang J.H., Huang S.L., Li J.X., Tian K., Liu Q. (2022). A Preliminary Study on the Effects of Combination of Biocontrol Agents Against Watermelon Fusarium Wilt Caused by *Fusarium oxysporum* f. sp. *niveum*. Guangdong Agric Sci.

[24] Huang Z.Y., Li Q.L., Gai X., Zhang X.P., Zhong Z.K., Bian F.Y. (2022). Corrigendum: effects of on- and off-year management practices on the soil organic C fractions and microbial community in a Moso bamboo (*Phyllostachys edulis*) forest in subtropical China. Front Plant Sci.

[25] Magoč T., Salzberg S.L. (2011). FLASH: fast length adjustment of short reads to improve genome assemblies. Bioinformatics.

[26] Caporaso J.G., Kuczynski J., Stombaugh J., Bittinger K., Bushman F., Costello E.K. (2010). QIIME allows analysis of high-throughput community sequencing data. Nat Methods.

[27] Edgar R.C. (2013). UPARSE: highly accurate OTU sequences from microbial amplicon reads. Nat Methods.

[28] Quast C., Pruesse E., Yilmaz P., Gerken J., Schweer T., Yarza P. (2013). The SILVA ribosomal RNA gene database project: improved data processing and web-based tools. Nucleic Acids Res.

[29] Ley R.E., Turnbaugh P., Klein S., Gordon J.I. (2006). Microbial ecology: human gut microbes associated with obesity. Nature.

[30] Tobias G., Orkun S.S. (2014). Synthetic microbial communities. Curr Opin Microbiol.

[31] Xu X.M., Caja D., Adele P., Ákos T.K., Carlos L.A. (2025). Composing a microbial symphony: synthetic communities for promoting plant growth. Trends Microbiol.

[32] Hu J., Wei Z., Friman V., Gu S.H., Wang X.F., Eisenhauer N. (2016). Probiotic diversity enhances rhizosphere microbiome function and plant disease suppression. mBio.

[33] Niu B., Paulson J.N., Zheng X.Q., Kolter R. (2017). Simplified and representative bacterial community of maize roots. Proc Natl Acad Sci U S A.

[34] Zhuang L.B., Li Y., Wang Z.S., Yu Y., Zhang N., Yang C. (2020). Synthetic community with six *Pseudomonas* strains screened from garlic rhizosphere microbiome promotes plant growth. Microb Biotechnol.

[35] Zareen K., Hyungmin R., Andrea F., Shang H.H., Virginia L., Oscar M. (2016). Growth enhancement and drought tolerance of hybrid poplar upon inoculation with endophyte consortia. Curr Plant Biol.

[36] Mitter B., Pfaffenbichler N., Flavell R., Compant S., Antonielli L., Petric A. (2017). A new approach to modify plant microbiomes and traits by introducing beneficial bacteria at flowering into progeny seeds. Front Microbiol.

[37] Günter B., Stéphane C., Kathryn V., Birgit M., Friederike T., Ma L.J. (2017). Ecology and genomic insights into plant-pathogenic and plant-nonpathogenic endophytes. Annu Rev Phytopathol.

[38] Berendsen R.L., Vismans G., Yu K., Song Y., de Jonge R., Burgman W.P. (2018). Disease-induced assemblage of a plant-beneficial bacterial consortium. ISME J.

[39] Mendes L.W., Mendes R., Raaijmakers J.M., Tsai S.M. (2018). Breeding for soil-borne pathogen resistance impacts active rhizosphere microbiome of common bean. ISME J.

[40] Jiang Y., Song Y., Jiang C.Y., Li X., Liu T.T., Wang J.R. (2022). Identification and characterization of *Arthrobacter nicotinovorans* JI39, a novel plant growth-promoting rhizobacteria strain from *Panax ginseng*. Front Plant Sci.

[41] Thomloudi E., Tsalgatidou P.C., Douka D., Spantidos T.N., Dimou M., Venieraki A. (2019). Multistrain versus single-strain plant growth promoting microbial inoculants - the compatibility issue. Hell Plant Prot J.

[42] Qu Q., Zhang Z.Y., Peijnenburg W., Liu W.Y., Lu T., Hu B.L. (2020). Rhizosphere microbiome assembly and its impact on plant growth. J Agric Food Chem.

[43] Hamid M.I., Hussain M., Wu Y.P., Zhang X.L., Xiang M.C., Liu X.Z. (2017). Successive soybean-monoculture cropping assembles rhizosphere microbial communities for the soil suppression of soybean cyst nematode. FEMS Microbiol Ecol.

[44] Hassani M.A., Durán P., Hacquard S. (2018). Microbial interactions within the plant holobiont. Microbiome.

[45] Dong L.L., Xu J., Zhang L.J., Cheng R.Y., Wei G.F., Su H. (2018). Rhizospheric microbial communities are driven by *Panax ginseng* at different growth stages and biocontrol bacteria alleviates replanting mortality. Acta Pharm Sin B.

[46] Zhang X.Y., Kong D., Liu X.Y., Xie H.H., Lou X.Y., Zeng C. (2021). Combined microbial degradation of crude oil under alkaline conditions by *Acinetobacter baumannii* and *Talaromyces* sp. Chemosphere.

[47] Atakpa E.O., Zhou H.H., Jiang L.J., Ma Y.H., Liang Y.P., Li Y.H. (2022). Improved degradation of petroleum hydrocarbons by co-culture of fungi and biosurfactant-producing bacteria. Chemosphere.

[48] Mariutto M., Duby F., Adam A., Bureau C., Fauconnier M.L., Ongena M. (2011). The elicitation of a systemic resistance by *Pseudomonas putida* BTP1 in tomato involves the stimulation of two lipoxygenase isoforms. BMC Plant Biol.

[49] Miltiadis V.C., Eleni T. (2015). Participation of phenylalanine ammonia-lyase (PAL) in increased phenolic compounds in fresh cold stressed walnut (*Juglans regia* L.) kernels. Postharvest Biol Technol.

[50] Bereika F.M., Nashwa M.S., Saad A.M., Kamal A.M., Yasser S.M. (2020). Mostafa; approving the biocontrol method of potato wilt caused by *Ralstonia solanacearum* (Smith) using *Enterobacter cloacae* PS14 and *Trichoderma asperellum* T34. Egy J Biol Pest Control.

[51] Chowdappa P., Mohan K., Lakshmi J.M., Upreti K.K. (2013). Growth stimulation and induction of systemic resistance in tomato against early and late blight by *Bacillus subtilis* OTPB1 or *Trichoderma harzianum* OTPB3. Biol Control.

[52] Wang S., Wu H.J., Qiao J.Q., Ma L.L., Liu J., Xia Y.F. (2009). Molecular mechanism of plant growth promotion and induced systemic resistance to tobacco mosaic virus by *Bacillus* spp. J Microbiol Biotechnol.

[53] Zhu Y.H., Shao Y.Y., Li L., Zhao L., Zhang M.J., Dong C.M. (2022). The plant growth-promoting endophytic *Fusarium oxysporum* GG22 enhances *Rehmannia glutinosa* secondary metabolites accumulation. Ind Crops Prod.

