# Supplementary material for: Synergistic interactions in designed bacterial consortia: Dual effects on control rusty root rot and growth promotion in ginseng
Source: Synth Syst Biotechnol. 2026 Jun 3;14:417–28. doi: 10.1016/j.synbio.2026.04.003 (PMC13265649; doi:10.1016/j.synbio.2026.04.003)
Supplement: Multimedia component 1 [file mmc1.docx]

Tab. S1 The biocontrol strains and pathogen strains in this test

| Biocontrol strains | | Biocontrol strains | | Diseases | Pathogens |
| --- | --- | --- | --- | --- | --- |
| NT35 | *Bacillus velezensis* | TJ23-2 | 1. *subtilis* | Ginseng rusty root rot | *Ilyonectria robusta* |
| NJ13 | 1. *methylotrophicus* | JJ5-2 | *Bacillus* sp. | Ginseng black disease | *Alternaria panax* |
| FG14 | *B. amyloliquefaciens* | JI39-2 | *Arthrobacter nicotinovorans* | Ginseng anthracnose | *Colletotrichum panacicola* |
| JA26 | *Acinetobacter* sp. | JI6 | *Pseudomonas thivervalensis* | Sclerotium blight of sunflower | *Sclerotinia sclerotiorum* |
| JA38 | *Burkholderia cepacia* |  |  | Tobacco root rot | *Fusarium oxysporum* |
| TU26 | *Pseudomonas chlororaphis* |  |  | Ginseng root rot | *Fusarium solani* |
|  |  |  |  | Bakanae of rice | *Fusarium moniliforme* |
|  |  |  |  | Watermelon stem blight | *Stagonosporopsis citrulli* |

Tab. S2 Medium formula

| Medium | Component & content |
| --- | --- |
| Luria-Bertani（LB） | Peptone 10.0 g/L, yeast extract 5.0 g/L，NaCl 10.0 g/L |
| LA | Peptone 10.0 g/L, yeast extract 5.0 g/L，NaCl 10.0 g/L，Agar 20.0 g/L |
| PD | Potato 20.0 g/L, glucose 18.0-20.0 g/L |
| PDA | Potato 20.0 g/L, glucose 18.0-20.0 g/L，Agar 15.0 g/L |
| Fermentation medium of strain NT35 | Sugar 15.0 g/L, yeast extract 25.0 g/L, ammonium sulfate 25.0 g/L, dipotassium hydrogen phosphate 15.0 g/L |
| Fermentation medium of strain TJ23-2 | Sodium chloride 6.0 g/L, glucose 10.0 g/L, magnesium sulfate 0.1 g/L, yeast extract 10.0 g/L |
| Fermentation medium of strain JJ5-2 | Sodium chloride 5.0 g/L, yeast extract 10.0 g/L, magnesium sulfate 0.8 g/L, glucose 10.0 g/L |
| Fermentation medium of strain FG14 | Sugar 5.2 g/L, ammonium oxalate 5.5 g/L, dipotassium hydrogen phosphate 3.3 g/L, yeast extract 20.3 g/L |
| Fermentation medium of strain NJ13 | Glucose 30.0 g/L, starch 15.0 g/L, yeast extract 15.0 g/L, dipotassium hydrogen phosphate 1.0 g/L, sodium chloride 5.0 g/L |
| Fermentation medium of strain JA38 | NaCl 9.0 g/L, beef extract 10.0 g/L, sodium citrate 10.0 g/L, ferrous sulfate 1.5 g/L. |
| Fermentation medium of strain JI39-2 | Potassium nitrate 17.5 g/L, magnesium sulfate 0.8 g/L, sodium chloride 15.0 g/L, glucose 20.0 g/L |
| Fermentation medium of strain JI6 | Yeast extract 19.9 g/L, potassium chloride 3.2 g/L, magnesium sulfate 1.1 g/L, sodium citrate 17.9 g/L |
| Fermentation medium of strain TU26 | Sodium chloride 5.0 g/L，yeast extract 10.0 g/L, magnesium sulfate 0.8 g/L, glucose 10.0 g/L |
| Fermentation medium of strain JA26 | Sodium chloride 5.0 g/L，yeast extract 10.0 g/L, magnesium sulfate 0.8 g/L, glucose 10.0 g/L |
| Ashby | Sucrose 10.0 g/L, potassium dihydrogen phosphate 0.2 g/L, magnesium sulfate heptahydrate 0.2 g/L, sodium chloride 0.2 g/L, calcium carbonate 5.0 g/L, calcium sulfate dihydrate 0.1 g/L, agar powder 15.0 g/L. |
| Pikovaskaia’s | Tricalcium phosphate 4.0 g/L, sucrose 10.0 g/L, ammonium sulfate 0.5 g/L, sodium chloride 0.2 g/L, magnesium sulfate heptahydrate 0.1 g/L, potassium chloride 0.2 g/L, yeast powder 0.05 g/L, sulfuric acid 0.1 mL (0.04 g / L), ferrous sulfate 0.1 mL (0.02 g / L), agar powder 15.0 g/L. |
| Silicate bacterial culture medium | Potassium feldspar powder 5.0 g/L, disodium hydrogen phosphate 2.0 g/L, calcium carbonate 0.1 g/L, magnesium sulfate heptahydrate 0.5 g/L, ferric chloride 0.5 g/L, sucrose 5.0 g/L, trehalose 1.0 g/L, agar powder 18.0 g/L. |

Tab. S3 Full combination of synergistic biocontrol bacteria

| Name of flora | Richness | Bacterial mixtures |
| --- | --- | --- |
| CL1 | 1 | NJ13 |
| CL2 | 1 | TU26 |
| CL3 | 1 | JJ5-2 |
| CL4 | 1 | TJ23-2 |
| CL5 | 1 | JI39-2 |
| CL6 | 1 | JI6 |
| CL7 | 1 | JA38 |
| CL8 | 1 | FG14 |
| CL9 | 1 | NT35 |
| CL10 | 1 | JA26 |
| CL11 | 2 | NJ13+TJ23-2 |
| CL12 | 2 | NJ13+JA38 |
| CL13 | 2 | NJ13+NT35 |
| CL14 | 2 | NT35+FG14 |
| CL15 | 2 | TJ23-2+JJ5-2 |
| CL16 | 2 | NJ13+FG14 |
| CL17 | 2 | JA38+JI6 |
| CL18 | 2 | NJ13+JI6 |
| CL19 | 2 | NJ13+TU26 |
| CL20 | 2 | TU26+JI6 |
| CL21 | 2 | JA38+JJ5-2 |
| CL22 | 2 | NJ13+JJ5-2 |
| CL23 | 2 | NJ13+JI39-2 |
| CL24 | 2 | TJ23-2+JI39-2 |
| CL25 | 2 | TU26+JI39-2 |
| CL26 | 2 | JJ5-2+JI39-2 |
| CL27 | 2 | JI6+JI39-2 |
| CL28 | 2 | FG14+JA26 |
| CL29 | 2 | NJ13+JA26 |
| CL30 | 2 | NT35+JA26 |
| CL31 | 2 | TU26+JA26 |
| CL32 | 2 | JI6+JA26 |
| CL33 | 2 | JA38+TU26 |
| CL34 | 2 | NT35+TU26 |
| CL35 | 2 | FG14+TU26 |
| CL36 | 2 | NT35+JI6 |
| CL37 | 2 | FG14+JI6 |
| CL38 | 2 | NJ13+JJ5-2 |
| CL39 | 2 | TU26+JJ5-2 |
| CL40 | 2 | JI6+JJ5-2 |
| CL41 | 2 | TJ23-2+TU26 |
| CL42 | 2 | TJ23-2+JI6 |
| CL43 | 3 | JA38+TU26+JI6 |
| CL44 | 3 | FG14+NT35+NJ13 |
| CL45 | 3 | NJ13+JJ5-2+TU26 |
| CL46 | 3 | NJ13+JJ5-2+JA38 |
| CL47 | 3 | NJ13+JI39-2+JI6 |
| CL48 | 3 | TJ23-2+JJ5-2+JI39-2 |
| CL49 | 3 | TJ23-2+JJ5-2+JI6 |
| CL50 | 3 | TJ23-2+TU26+JI6 |
| CL51 | 3 | NJ13+TU26+JI39-2 |
| CL52 | 3 | JI6+JI39-2+TJ23-2 |
| CL53 | 3 | NJ13+TU26+JA38 |
| CL54 | 3 | NJ13+TJ23-2+JI39-2 |
| CL55 | 3 | NJ13+TU26+JJ5-2 |
| CL56 | 3 | TJ23-2+JI6+JJ5-2 |
| CL57 | 3 | NJ13+TJ23-2+JI6 |
| CL58 | 3 | NJ13+TU26+JI6 |
| CL59 | 3 | NJ13+JA38+JJ5-2 |
| CL60 | 3 | FG14+NT35+JA26 |
| CL61 | 3 | FG14+NJ13+JA26 |
| CL62 | 3 | NT35+NJ13+JA26 |
| CL63 | 3 | FG14+NJ13+TU26 |
| CL64 | 3 | FG14+NT35+TU26 |
| CL65 | 3 | NJ13+NT35+TU26 |
| CL66 | 3 | FG14+NJ13+JI6 |
| CL67 | 3 | FG14+NT35+JI6 |
| CL68 | 3 | NJ13+NT35+JI6 |
| CL69 | 4 | NJ13+JJ5-2+JA38+TU26 |
| CL70 | 4 | NJ13+TU26+JJ5-2+JI6 |
| CL71 | 4 | TJ23-2+NJ13+JI39-2+JI6 |
| CL72 | 4 | TJ23-2+NJ13+JI39-2+TU26 |
| CL73 | 4 | JJ5-2+NJ13+JI39-2+TU26 |
| CL74 | 4 | JJ5-2+NJ13+JI39-2+JI6 |
| CL75 | 4 | TJ23-2+JI6+JJ5-2+TU26 |
| CL76 | 4 | TJ23-2+JI6+JI39-2+TU26 |
| CL77 | 4 | FG14+NJ13+NT35+TU26 |
| CL78 | 4 | NJ13+FG14+NT35+JA26 |
| CL79 | 4 | JA38+JJ5-2+TU26+JI6 |
| CL80 | 4 | JJ5-2+NJ13+TU26+JI6 |
| CL81 | 4 | NJ13+FG14+NT35+JI6 |
| CL82 | 4 | FG14+NT35+TU26+JA26 |
| CL83 | 5 | FG14+NT35+TU26+JI6+JA26 |
| CL84 | 5 | NJ13+NT35+TU26+JI6+JA26 |
| CL85 | 5 | NJ13+FG14+TU26+JI6+JA26 |
| CL86 | 5 | NJ13+TJ23-2+JJ5-2+TU26+JI39-2 |
| CL87 | 5 | NJ13+TJ23-2+JJ5-2+JI39-2+JI6 |
| CL88 | 5 | NJ13+TJ23-2+JJ5-2+TU26+JI6 |
| CL89 | 5 | NJ13+JJ5-2+JI39-2+TU26+JI6 |
| CL90 | 5 | NJ13+NT35+FG14+JI6+JA26 |
| CL91 | 5 | TJ23-2+JI6+JJ5-2+TU26+JI39-2 |
| CL92 | 5 | NJ13+TU26+JI6+TJ23-2+JI39-2 |
| CL93 | 5 | NJ13+TU26+JJ5-2+JI6+TJ23-2 |
| CL94 | 5 | NJ13+TU26+JJ5-2+JI6+JA38 |
| CL95 | 5 | NJ13+NT35+FG14+TU26+JI6 |
| CL96 | 5 | NJ13+NT35+FG14+TU26+JA26 |
| CL97 | 6 | NJ13+TJ23-2+JJ5-2+JI39-2+TU26+JI6 |
| CL98 | 6 | NJ13+FG14+NT35+TU26+JI6+JA26 |

Tab. S4 The prevention effect for disease by using bacterial group combinations

| Number | | Richness | | Bacterial mixtures | | Combinations | | Spot size/mm | | Spot depth/mm | |
| --- | --- | --- | --- | --- | --- | --- | --- | --- | --- | --- | --- |
| 1 | | 1 | | NJ13 | | CL1 | | 6.50±0.341 d | | 6.50±0.224 d | |
| 2 | | 1 | | TU26 | | CL2 | | 7.00±0.421 c | | 8.00±0.336 b | |
| 3 | | 1 | | JJ5-2 | | CL3 | | 7.55±0.441 b | | 7.55±0.336 c | |
| 4 | | 1 | | TJ23-2 | | CL4 | | 7.07±0.166 d | | 7.47±0.247 c | |
| 5 | | 1 | | JI39-2 | | CL5 | | 7.33±0.332 bc | | 8.33±0.653 ab | |
| 6 | | 1 | | JI6 | | CL6 | | 7.48±0.857 b | | 8.48±0.421 a | |
| 7 | | 1 | | JA38 | | CL7 | | 6.44±0.518 d | | 7.00±0.422 cd | |
| 8 | | 1 | | FG14 | | CL8 | | 7.25±0.422 bc | | 7.08±0.832 cd | |
| 9 | | 1 | | NT35 | | CL9 | | 7.08±0.283 c | | 6.75±0.533 d | |
| 10 | | 1 | | JA26 | | CL10 | | 8.17±0.321 a | | 7.75±0.671 bc | |
| 11 | | 2 | | NJ13+TJ23-2 | | CL11 | | 5.77±0.353 f | | 5.86±0.553 e | |
| 12 | | 2 | | NJ13+JA38 | | CL12 | | 5.83±0.806 e | | 5.75±0.324 e | |
| 13 | | 2 | | NJ13+NT35 | | CL13 | | 5.83±0.243 e | | 5.67±0.853 e | |
| 14 | | 2 | | NT35+FG14 | | CL14 | | 5.67±0.231 e | | 5.43±0.561 e | |
| 15 | | 2 | | TJ23-2+JJ5-2 | | CL15 | | 5.47±0.633 e | | 5.58±0.734 e | |
| 16 | | 2 | | NJ13+FG14 | | CL16 | | 5.67±0.133 e | | 5.55±0.763 e | |
| 17 | | 3 | | JA38+TU26+JI6 | | CL43 | | 3.00±0.217 h | | 3.00±0.477 h | |
| 18 | | 3 | | FG14+NT35+NJ13 | | CL44 | | 4.87±0.233 ef | | 5.65±0.773 e | |
| 19 | | 3 | | NJ13+JJ5-2+TU26 | | CL45 | | 5.00±0.623 ef | | 5.05±0.353 f | |
| 20 | | 3 | | NJ13+JJ5-2+JA38 | | CL46 | | 5.27±0.358 ef | | 5.15±0.255 f | |
| 21 | | 4 | | NJ13+JJ5-2+JA38+TU26 | | CL69 | | 3.83±0.133 h | | 3.83±0.261 h | |
| 22 | | 4 | | TJ23-2+NJ13+JI39-2+JI6 | | CL71 | | 5.00±0.413 ef | | 5.25±0.363 f | |
| 23 | | 4 | | TJ23-2+NJ13+JI39-2+TU26 | | CL72 | | 4.25±0.455 fg | | 5.25±0.725 f | |
| 24 | 4 | | JJ5-2+NJ13+JI39-2+TU26 | | CL73 | | 4.33±0.554 f | | 5.21±0.621 f | |  |
| 25 | 4 | | JJ5-2+NJ13+JI39-2+JI6 | | CL74 | | 4.33±0.455 f | | 5.04±0.833 f | |  |
| 26 | 4 | | TJ23-2+JI6+JJ5-2+TU26 | | CL75 | | 4.15±0.342 g | | 5.00±0.638 fg | |  |
| 27 | 4 | | TJ23-2+JI6+JI39-2+TU26 | | CL76 | | 4.00±0.233 g | | 4.50±0.492 g | |  |
| 28 | 4 | | FG14+NJ13+NT35+TU26 | | CL77 | | 4.00±0.335 g | | 4.30±0.482 g | |  |
| 29 | 4 | | NJ13+FG14+NT35+JA26 | | CL78 | | 4.18±0.338 g | | 4.00±0.655 g | |  |
| 30 | 4 | | JA38+JJ5-2+TU26+JI6 | | CL79 | | 4.17±0.233 g | | 4.45±0.759 g | |  |
| 31 | 4 | | JJ5-2+NJ13+TU26+JI6 | | CL80 | | 4.75±0.166 ef | | 4.75±0.521 fg | |  |
| 32 | 4 | | FG14+NT35+NJ13+JI6 | | CL81 | | 5.00±0.234 ef | | 5.00±0.804 f | |  |
| 33 | 5 | | TJ23-2+JI6+JJ5-2+TU26+JI39-2 | | CL91 | | 2.83±0.433 i | | 3.85±0.6713 h | |  |
| 34 | 5 | | NJ13+TU26+JI6+TJ23-2+JI39-2 | | CL92 | | 3.50±0.334 h | | 4.50±0.725 g | |  |
| 35 | 5 | | NJ13+TU26+JJ5-2+JI6+TJ23-2 | | CL93 | | 3.58±0.122 h | | 4.58±0.783 fg | |  |
| 36 | 5 | | NJ13+TU26+JJ5-2+JI6+JA38 | | CL94 | | 2.00±0.224 j | | 2.03±0.361 j | |  |
| 37 | 5 | | NJ13+NT35+FG14+TU26+JI6 | | CL95 | | 3.47±0.566 h | | 3.33±0.136 hi | |  |
| 38 | 5 | | NJ13+NT35+FG14+TU26+JA26 | | CL96 | | 3.25±0.258 h | | 3.00±0.168 i | |  |
| 39 | 6 | | NJ13+TJ23-2+JJ5-2+JI39-2+TU26+JI6 | | CL97 | | 2.08±0.346 j | | 2.00±0.667 j | |  |
| 40 | 6 | | NJ13+FG14+NT35+TU26+JI6+JA26 | | CL98 | | 2.00±0.286 j | | 1.67±0.106 j | |  |

Tab. S5 The growth-promoting effect by biocontrol flora

| No. | Richness | Bacterial mixtures | Combinations | Nitrogen fixation /mm | Melting phosphrous  /mm | Potassium releasing  /mm |
| --- | --- | --- | --- | --- | --- | --- |
| 1 | 1 | NJ13 | CL1 | - | - | 2.67±0.344k |
| 2 | 1 | TU26 | CL2 | 6.00±0.235g | 2.67±0.333h | 5.67±0.267j |
| 3 | 1 | JJ5-2 | CL3 | - | - | 2.00±0.153k |
| 4 | 1 | TJ23-2 | CL4 | - | - | 2.00±0.633k |
| 5 | 1 | JI39-2 | CL5 | - | - | 4.00±0.353jk |
| 6 | 1 | JI6 | CL6 | 6.00±0.322g | 7.00±0.225g | 7.33±0.553i |
| 7 | 1 | JA38 | CL7 | 8.00±0.355g | 9.00±0.167f | 7.67±0.133h |
| 8 | 1 | FG14 | CL8 | - | 4.00±0.056h | 2.00±0.132k |
| 9 | 1 | NT35 | CL9 | - | 4.00±0.455h | 2.67±0.167k |
| 10 | 1 | JA26 | CL10 | - | 4.67±0.422h | 6.00±0.347i |
| 11 | 2 | NJ13+TJ23-2 | CL11 | - | - | 12.00±0.241g |
| 12 | 2 | NJ13+JA38 | CL12 | 8.00±0.322g | 12.00±0.455d | 13.00±0.224g |
| 13 | 2 | NJ13+NT35 | CL13 | - | 10.00±0.234f | 12.00±0.324g |
| 14 | 2 | NT35+FG14 | CL14 | - | 10.00±0.518f | 12.00±0.253g |
| 15 | 3 | JA38+TU26+JI6 | CL43 | 14.00±0.463d | 14.33±0.387c | 22.00±0.477b |
| 16 | 3 | NJ13+NT35+FG14 | CL44 | - | 13.00±0.147d | 14.00±0.281f |
| 17 | 4 | NJ13+TU26+JJ5-2+JA38 | CL69 | 15.00±0.476c | 15.83±0.224b | 18.00±0.341e |
| 18 | 4 | TJ23-2+JI6+JJ5-2+TU26 | CL75 | 15.00±0.255c | 16.16±0.324b | 20.67±0.445d |
| 19 | 4 | TJ23-2+JI6+JI39-2+TU26 | CL76 | 15.17±0.267c | 16.33±0.133b | 20.50±0.355d |
| 20 | 4 | JA38+JJ5-2+TU26+JI6 | CL79 | 15.17±0.166c | 14.67±0.083c | 20.00±0.271d |
| 21 | 4 | NJ13+TU26+JJ5-2+JI6 | CL80 | 16.00±0.133b | 15.33±0.185b | 21.00±0.188c |
| 22 | 5 | TJ23-2+JI6+JJ5-2+TU26+JI39-2 | CL91 | 16.33±0.235b | 17.00±0.477b | 23.17±0.167b |
| 23 | 5 | NJ13+TU26+JI6+TJ23-2+JI39-2 | CL92 | 16.00±0.271b | 16.83±0.255b | 22.00±0.233c |
| 24 | 5 | NJ13+TU26+JJ5-2+JI6+TJ23-2 | CL93 | 16.17±0.447b | 17.17±0.267b | 22.83±0.342bc |
| 25 | 5 | NJ13+TU26+JJ5-2+JI6+JA38 | CL94 | 16.50±0.244b | 16.67±0.355b | 25.00±0.277b |
| 26 | 5 | NJ13+NT35+FG14+TU26+JI6 | CL95 | 13.00±0.253e | 15.50±0.133c | 19.00±0.431d |
| 27 | 5 | NJ13+NT35+FG14+TU26+JA26 | CL96 | 12.00±0.258e | 16.33±0.324b | 19.00±0.341d |
| 28 | 6 | NJ13+TU26+JJ5-2+JI6+TJ23-2+JI39-2 | CL97 | 17.83±0.463a | 18.83±0.255a | 30.00±0.477a |
| 29 | 6 | NJ13+NT35+FG14+TU26+JA26+JI6 | CL98 | 14.00±0.422d | 22.00±0.541a | 23.00±0.246b |

Tab. S6 Colonizing flora of the whole combination of compound

| Combination | Richness | Bacterial treatment | | | | | | | | | |
| --- | --- | --- | --- | --- | --- | --- | --- | --- | --- | --- | --- |
|  |  | TJ23-2 | JJ5-2 | NT35 | NJ13 | FG14 | JA26 | JA38 | JI39-2 | TU26 | JI6 |
| 1 | 1 | 0 | 0 | 1 | 0 | 0 | 0 | 0 | 0 | 0 | 0 |
| 2 | 1 | 0 | 0 | 0 | 1 | 0 | 0 | 0 | 0 | 0 | 0 |
| 3 | 1 | 0 | 0 | 0 | 0 | 1 | 0 | 0 | 0 | 0 | 0 |
| 4 | 2 | 0 | 0 | 1 | 1 | 0 | 0 | 0 | 0 | 0 | 0 |
| 5 | 3 | 0 | 0 | 1 | 1 | 1 | 0 | 0 | 0 | 0 | 0 |
| 6 | 5 | 1 | 0 | 0 | 1 | 0 | 0 | 0 | 1 | 1 | 1 |
| 7 | 5 | 0 | 0 | 1 | 1 | 1 | 0 | 0 | 0 | 1 | 1 |
| 8 | 6 | 1 | 1 | 0 | 1 | 0 | 0 | 0 | 1 | 1 | 1 |

Note: 1 and 0 indicate whether the strain is or is not involved in the construction of the complex flora.

Tab. S7 the test of soil enzyme activity by combination of biocontrol flora

| Combination | Richness | Bacterial treatment | | | | | | | | | | |
| --- | --- | --- | --- | --- | --- | --- | --- | --- | --- | --- | --- | --- |
|  |  | TJ23-2 | JJ5-2 | NT35 | NJ13 | FG14 | JA26 | JA38 | JI39-2 | TU26 | JI6 |  |
| 1 | 1 | 0 | 0 | 1 | 0 | 0 | 0 | 0 | 0 | 0 | 0 |  |
| 2 | 1 | 0 | 0 | 0 | 1 | 0 | 0 | 0 | 0 | 0 | 0 |  |
| 3 | 1 | 0 | 0 | 0 | 0 | 1 | 0 | 0 | 0 | 0 | 0 |  |
| 4 | 2 | 0 | 0 | 1 | 1 | 0 | 0 | 0 | 0 | 0 | 0 |  |
| 5 | 3 | 0 | 0 | 1 | 1 | 1 | 0 | 0 | 0 | 0 | 0 |  |
| 6 | 4 | 0 | 1 | 0 | 0 | 0 | 0 | 1 | 0 | 1 | 1 |  |
| 7 | 5 | 0 | 0 | 1 | 1 | 1 | 1 | 0 | 0 | 1 | 0 |  |
| 8 | 5 | 1 | 0 | 0 | 1 | 0 | 0 | 0 | 1 | 1 | 1 |  |
| 9 | 5 | 0 | 0 | 1 | 1 | 1 | 0 | 0 | 0 | 1 | 1 |  |

Note: 1 and 0 indicate whether the strain is or is not involved in the construction of the complex flora.

Tab. S8 Real-time PCR detection information

| Genes | Accessing number | Primer name | Sequence ( 5 ' -3 ' ) | Tm/℃ |
| --- | --- | --- | --- | --- |
| *Phenylalanine ammonia-lyase* | DQ417194 | PAL-F | CAAAAGCTACATGAAATGGACCCT | 54 |
|  |  | PAL-R | GAGACATCAATCAATGGGTTATCG |  |
| *β-1.3 glucanase* | DQ015705 | Beta -F | GGGTAATCTTCCTTACGCATTG | 56 |
|  |  | Beta -R | GGCCATCCACTCTCCGACA |  |
| *Chitinase* | FJ790420 | Chi-F | CCTTCCGTCGTTTCGATATTTA | 54 |
|  |  | Chi-R | TACTAGCCTCATCCCTTGCAGT |  |
| *Superoxide Dismutase* | MF034869 | SOD-R | ATTGAGVCTGACGATCACAG | 60 |
|  |  | SOD-F | CCTTTGTCCCTTGCTTCTCTAG |  |
| *Peroxidase* | EU327037 | CAT-R | ATCCCAAGTCCCACATTCAG | 57 |
|  |  | CAT-F | ACATAGTGTGCTTTCCCTGC |  |
| *β-actin* | AY907207 | Act-F | TGCCCCAGAAGAGCACCCTGT | 62 |
|  |  | Act-R | AGCATACAGGGAAAGATCGGCTTGA |  |


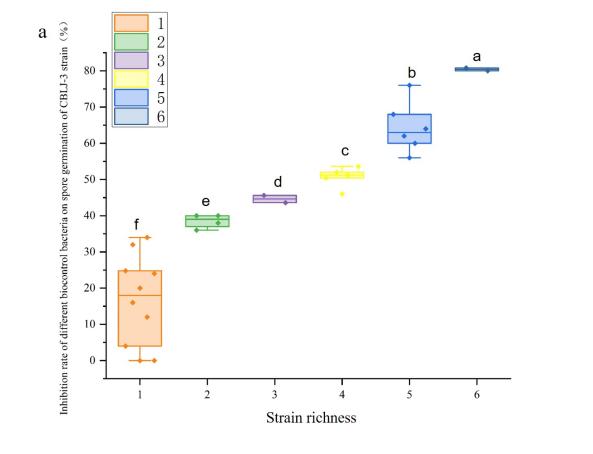

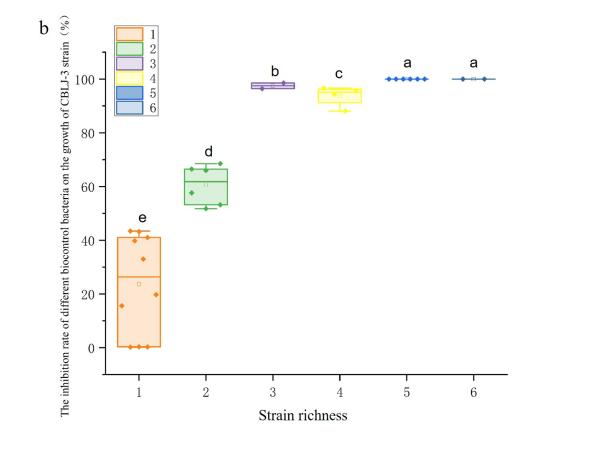


Fig. S1 Inhibitory effect of biocontrol bacteria on the spores (a) and mycelium (b) growth of CBLJ-3

Note: Different colors are combinations of different richness. The dots in the figure represent the effect of different bacterial combinations on the spores of pathogen.

Fig.S2 Inhibitory effect of different biocontrol bacteria and flora on plant pathogenic bacteria


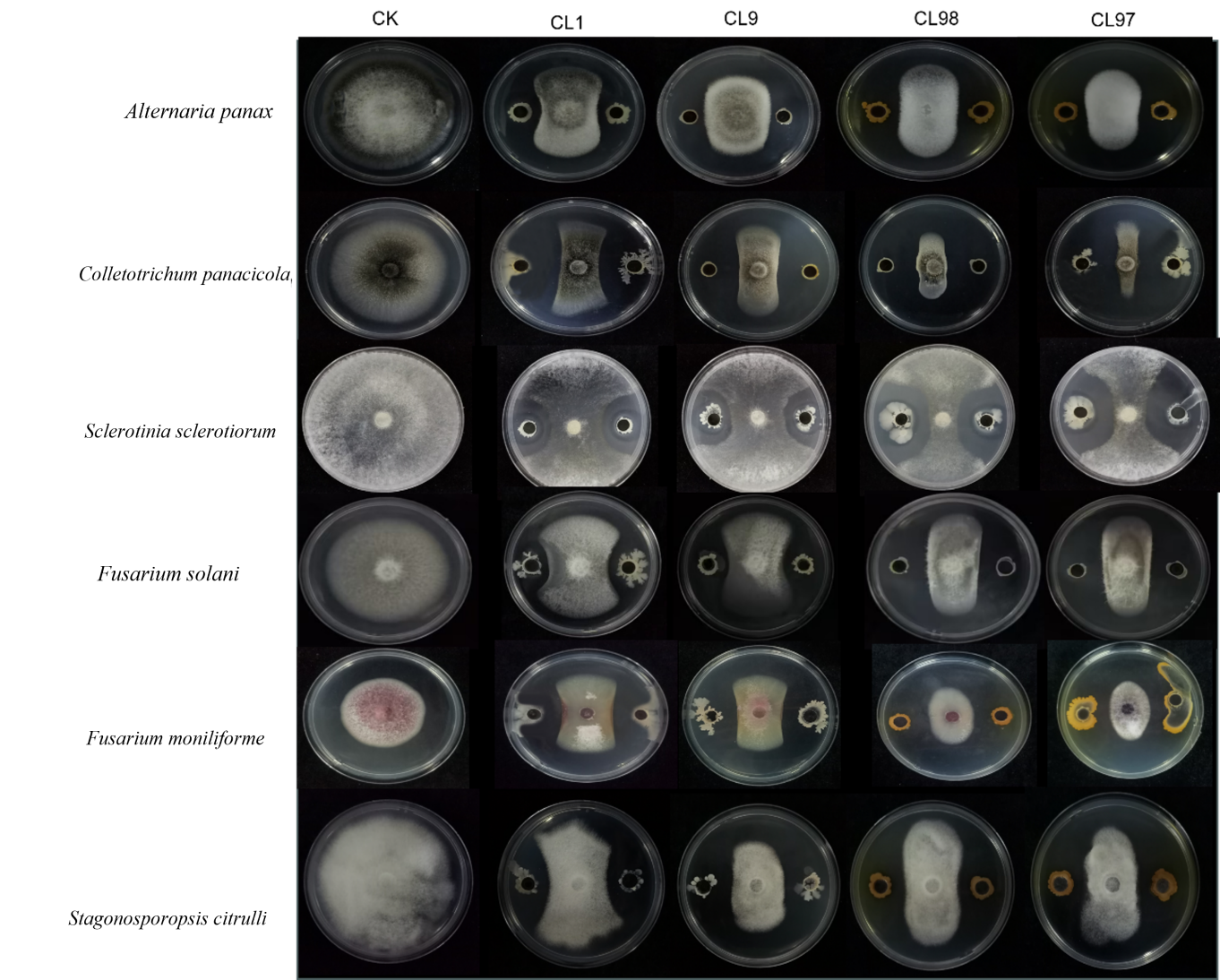

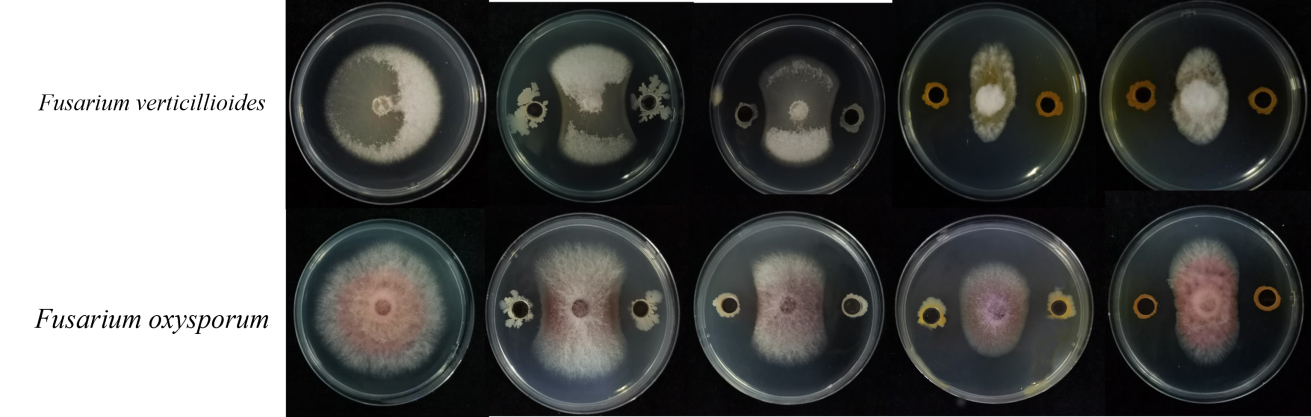

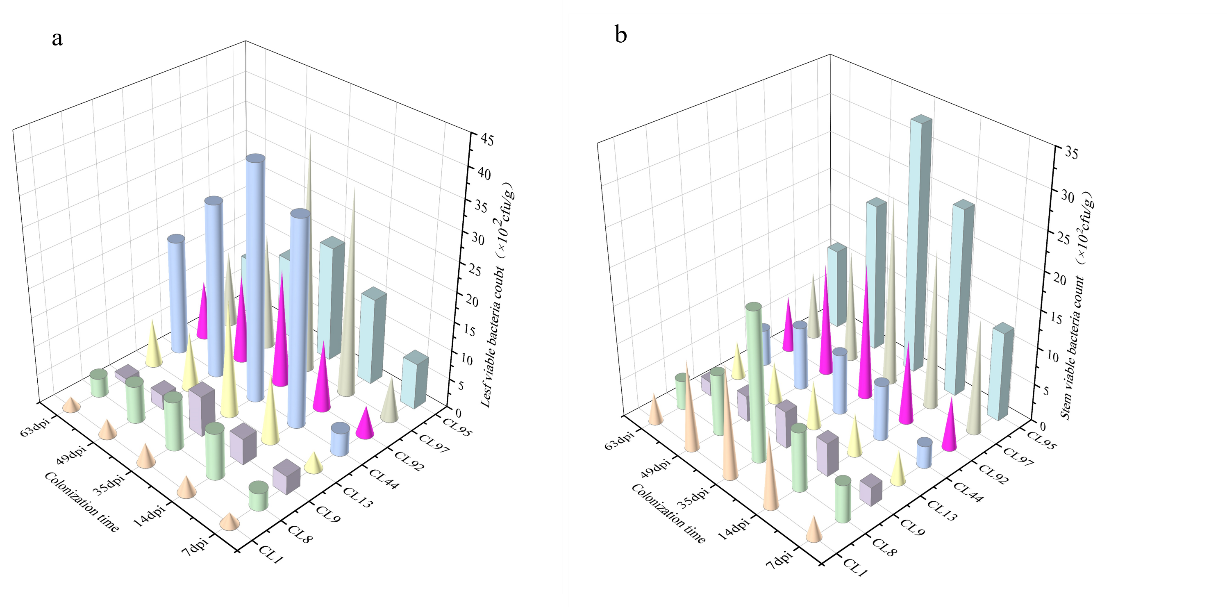

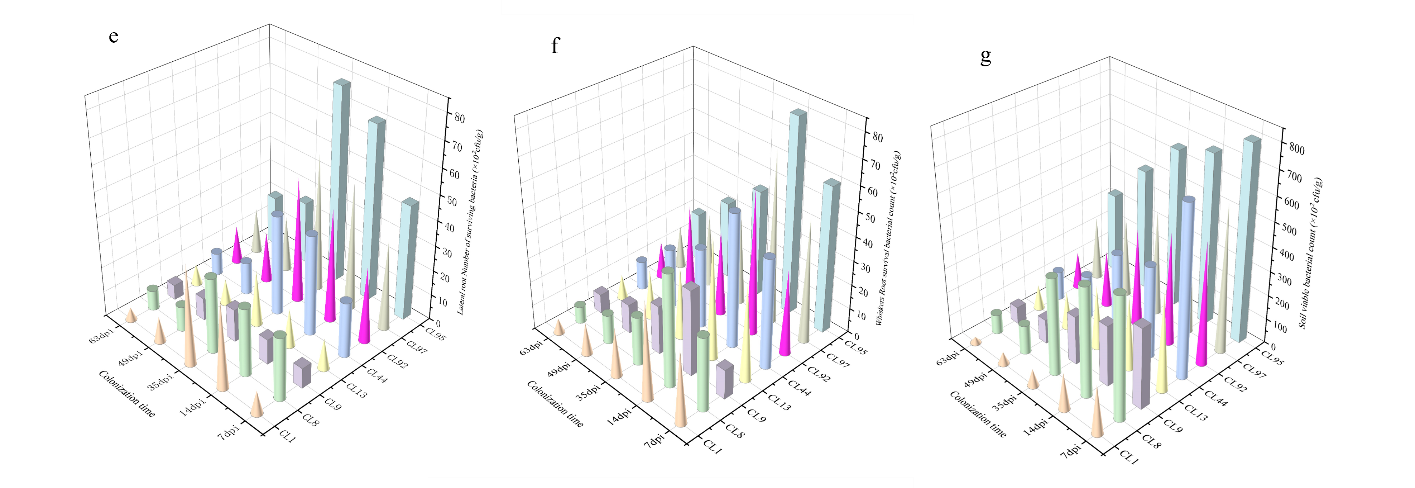

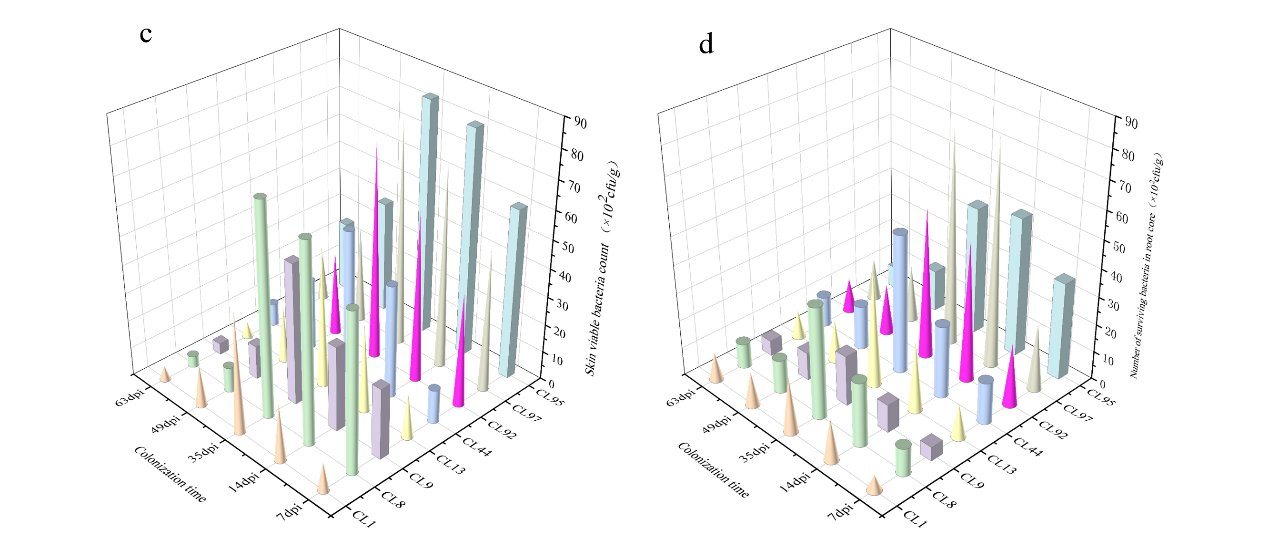


Fig. S3 Colonization changes of biocontrol single strain and flora in ginseng and rhizosphere soil

(a) leaf (b) stem (c) epidermis (d) root core (e) lateral root (f) fibrous root (g) rhizosphere soil

Note: Different colors are different richness flora combinations. The dots in the figure represent the antibacterial diameter of different flora combinations against ginseng rust rot pathogens.

Fig S4 Changes of microbial community diversity in ginseng rhizosphere soil

(a-c) Chao 1, Shannon and Simpson index of bacterial community; (d-f) Chao 1, Shannon and Simpson index of the fungal community.


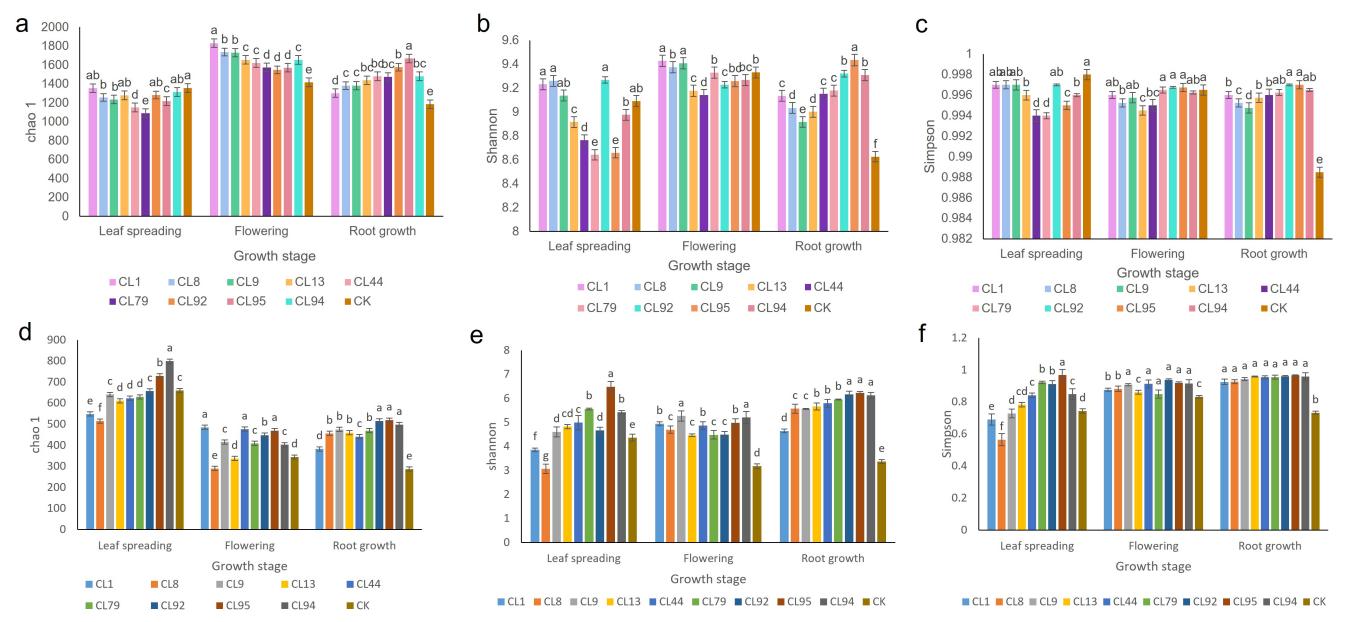

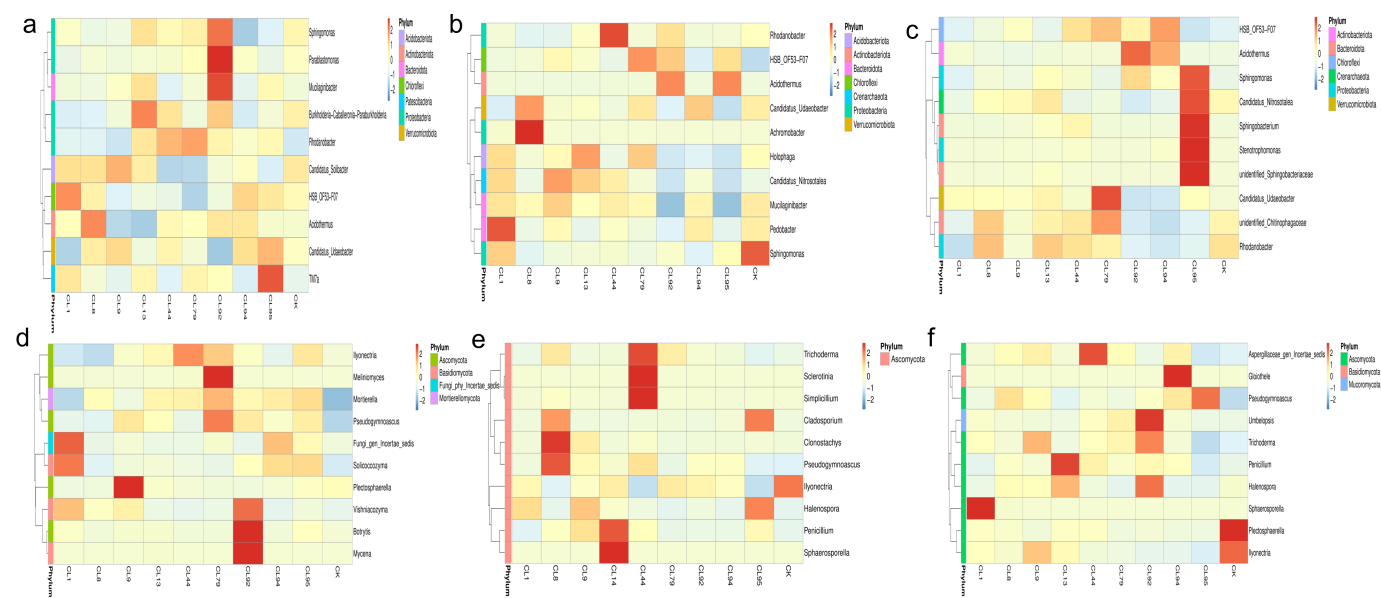


Fig S5 Composition of microbial groups at genus level in different growth stages of *Panax ginseng*

(a-c) Bacterial community composition; (d-f) Fungal community composition; (a, d) Leaf spreading; (b, e) Flowering; (c, f) Root growth


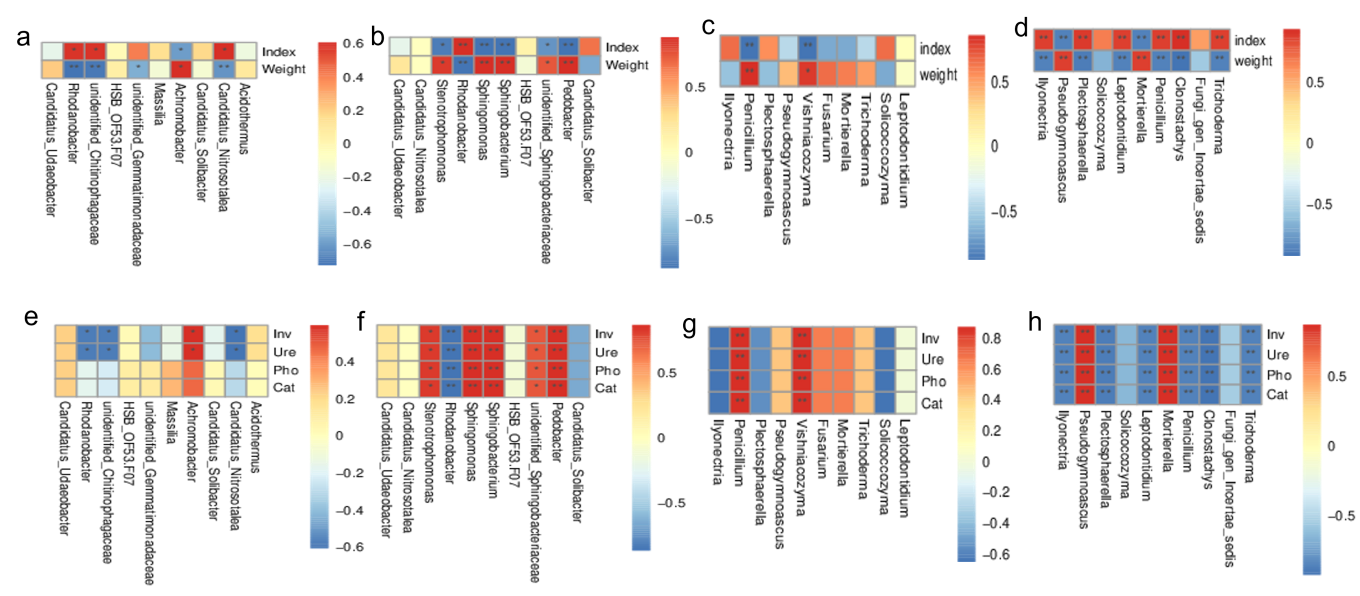


Fig S6 Associations between bacterial and fungal communities and relative factors

The correlation between bacterial microbial community and disease index (a-b), root growth and soil enzyme activity of ginseng (e-f); The correlation between fungal microbial community and ginseng disease index (c-d), root growth and soil enzyme activity (g-h).Note: Cat, catalase; Pho, alkaline phosphatase; Ure, urease; Inv, invertase. Index: disease index of ginseng rusty root rot; *p*-values were indicated by * symbol: ^**^*p* < 0.01; ^*^*p* < 0.05.
